# Efficacy and Safety of Bimekizumab in Patients With Psoriatic Arthritis With or Without Methotrexate: 52‐Week Results From Two Phase 3 Studies

**DOI:** 10.1002/acr2.11727

**Published:** 2024-07-30

**Authors:** Iain B. McInnes, Philip J. Mease, Yoshiya Tanaka, Laure Gossec, M. Elaine Husni, Lars Erik Kristensen, Richard B. Warren, Barbara Ink, Rajan Bajracharya, Jason Coarse, Alice B. Gottlieb

**Affiliations:** ^1^ College of Medical Veterinary and Life Sciences University of Glasgow Glasgow United Kingdom; ^2^ Swedish Medical Center/Providence St. Joseph Health and University of Washington Seattle; ^3^ University of Occupational and Environmental Health Japan, Kitakyushu Fukuoka Japan; ^4^ Sorbonne Université, INSERM, Institut Pierre Louis d'Epidémiologie et de Santé Publique and AP‐HP, Pitié‐Salpêtrière Hospital Paris France; ^5^ Cleveland Clinic Cleveland Ohio; ^6^ The Parker Institute, Copenhagen University Hospital, Bispebjerg and Frederiksberg Denmark; ^7^ Northern Care Alliance NHS Foundation Trust and NIHR Manchester Biomedical Research Centre, Manchester University NHS Foundation Trust, Manchester Academic Health Science Centre Manchester United Kingdom; ^8^ UCB Pharma Slough United Kingdom; ^9^ UCB Pharma Morrisville North Carolina; ^10^ The Icahn School of Medicine at Mount Sinai New York New York

## Abstract

**Objective:**

The objective of this study was to assess 52‐week efficacy and safety of bimekizumab in patients with active psoriatic arthritis (PsA) with or without concomitant methotrexate (+/−MTX) treatment at baseline.

**Methods:**

We conducted a post hoc analysis of patients in BE OPTIMAL (NCT03895203; biologic disease‐modifying antirheumatic drug [bDMARD]‐naïve), BE COMPLETE (NCT03896581; prior inadequate response or intolerance to tumor necrosis factor inhibitors [TNFi‐IR]), and the BE VITAL open‐label extension (NCT04009499) study. Patients were randomized to one of the following treatment groups: bimekizumab 160 mg every four weeks, placebo, or a reference drug (adalimumab 40 mg every two weeks; BE OPTIMAL only). From Week 16, placebo‐randomized patients received bimekizumab. Missing data were imputed using non‐responder imputation, multiple imputation, or worst‐category imputation.

**Results:**

Through Week 52, similar proportions of bimekizumab‐treated patients achieved American College of Rheumatology 50% (ACR50) response criteria for both +MTX and −MTX (BE OPTIMAL: 54.4% +MTX, 54.7% −MTX; BE COMPLETE: 56.3% +MTX, 48.0% −MTX). Similar proportions of bimekizumab‐treated patients achieved complete skin clearance (Psoriasis Area and Severity Index 100% [PASI100] response) and minimal disease activity in both +MTX and −MTX groups. Similar trends were seen in placebo/bimekizumab‐treated patients. Through Week 52, the proportion of bimekizumab‐treated patients with ≥1 treatment‐emergent adverse event were similar between the +MTX and −MTX groups (BE OPTIMAL 325 of 410 [79.3%] vs 230 of 292 [78.8%], BE COMPLETE 105 of 168 [62.5%] vs 138 of 220 [62.7%]). The safety profile was comparable between subgroups and consistent with the prior safety profile of bimekizumab.

**Conclusion:**

Treatment with bimekizumab demonstrated consistent, sustained efficacy to 52 weeks in bDMARD‐naïve and TNFi‐IR patients with PsA and was well tolerated, irrespective of concomitant MTX.

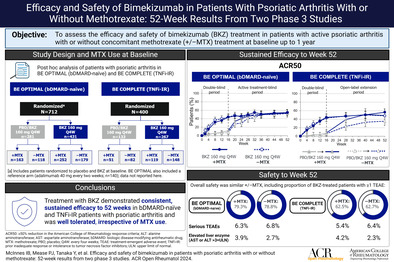

## INTRODUCTION

Psoriatic arthritis (PsA) is a progressive, long‐term, chronic inflammatory disease characterized by symptoms affecting a variety of tissues. PsA is heterogeneous, variably affecting peripheral and axial joints, skin, nails, and entheses.^1^, ^2^ Long‐term therapy is required, and multiple therapeutic options are now available.^3^, ^4^, ^5^ First‐line biologic therapies, such as tumor necrosis factor inhibitors (TNFi), are often given in combination with conventional synthetic disease‐modifying antirheumatic drugs, such as methotrexate (MTX).^6^ MTX treatment in PsA has typically been founded on evidence from other autoimmune diseases, such as rheumatoid arthritis and psoriasis, and is recommended within the PsA treatment guidelines despite a relative paucity of randomized controlled trial evidence for clinical effectiveness in this patient population.^3^, ^4^, ^7^, ^8^, ^9^, ^10^ The effect of MTX in combination with TNFi in the treatment of patients with PsA has been explored previously, without consensus on the added benefit it confers to the efficacy or retention on MTX. Some studies have found similar efficacy with or without concomitant MTX, albeit with an increased length of time before drug discontinuation when using MTX,^5^, ^6^, ^11^, ^12^ whereas others have reported increased remission rates with TNFi plus MTX combination therapy.^13^ Despite these studies, there is little evidence to support an added benefit of concomitant MTX with targeted therapy, such as biologic disease‐modifying antirheumatic drugs (bDMARDs), in patients with PsA with peripheral or skin disease, and no evidence of a benefit of MTX in patients with axial symptoms.^5^, ^9^

With the growing repertoire of bDMARD treatments available for patients with PsA, there is a need to understand the impact of concomitant MTX on efficacy and safety when used in combination with bDMARDs. Bimekizumab is a humanized monoclonal IgG1 antibody that selectively and potently inhibits interleukin (IL)‐17F in addition to IL‐17A. The efficacy and safety of bimekizumab have been reported up to one year from the BE OPTIMAL and BE COMPLETE/BE VITAL phase 3 studies of patients naïve to bDMARDs or with prior inadequate response or intolerance to TNFi (TNFi‐IR), respectively.^14^, ^15^

Analogous to prior evaluations of TNFi therapeutics, here we assess the clinical safety and efficacy of subcutaneous bimekizumab to Week 52 in bDMARD‐naïve and TNFi‐IR patients with PsA, with and without concomitant MTX (+/−MTX) at baseline.

## PATIENTS AND METHODS

### Study design and participants

The BE OPTIMAL (NCT03895203), BE COMPLETE (NCT03896581), and BE VITAL open‐label extension (OLE; NCT04009499) study designs have been reported previously.^15^, ^16^, ^17^ All studies assessed subcutaneous bimekizumab 160 mg every four weeks (Q4W) in patients with PsA.

In brief, BE OPTIMAL was a 52‐week, phase 3, multicenter, randomized, double‐blind, placebo‐controlled, active reference study of bDMARD‐naïve patients with PsA. The study comprised a 16‐week placebo‐controlled, double‐blind period, followed by a 36‐week active treatment‐blind period. Placebo‐randomized patients switched to receive bimekizumab 160 mg Q4W from Week 16. An adalimumab 40 mg every two weeks (Q2W) reference arm was included to provide a reference for the benefit/risk profile of bimekizumab alongside a standard‐of‐care treatment. This arm was not powered for statistical comparison to bimekizumab or placebo. Adalimumab reference arm data from BE OPTIMAL are reported in the Supplementary Material (Supplementary Tables S1–S3, Supplementary Figures S2–S4). Patients were randomized 3:2:1 to bimekizumab 160 mg Q4W or placebo or adalimumab 40 mg Q2W. All patients who completed Week 52 of BE OPTIMAL were eligible for enrollment into the BE VITAL OLE study and received bimekizumab 160 mg Q4W.

BE COMPLETE was a 16‐week, phase 3, multicenter, randomized, double‐blind, placebo‐controlled study of patients with active PsA and prior TNFi‐IR. Patients were randomized 2:1 to bimekizumab 160 mg Q4W or placebo. Those completing Week 16 of BE COMPLETE, meeting eligibility criteria, and providing separate informed consent were eligible for enrollment into the BE VITAL OLE study. All patients, including those randomized to placebo, received bimekizumab 160 mg Q4W during the BE VITAL OLE. Results are reported for patients randomized in BE COMPLETE and entering the BE VITAL OLE, hereafter referred to as BE COMPLETE only. Studies were conducted in accordance with the Declaration of Helsinki and the International Conference on Harmonization guidance for Good Clinical Practice. Ethical approval was obtained from the relevant institutional review boards at participating sites, and all patients provided written informed consent in accordance with local requirements.

### Patients

Inclusion and exclusion criteria have been reported previously.^14^, ^15^, ^16^, ^17^ Patients in BE OPTIMAL and BE COMPLETE were recruited during the same period of time at overlapping study sites. Patients had a documented diagnosis of adult‐onset PsA meeting the classification criteria for PsA for six or more months before screening.^18^ Patients had active PsA with a baseline tender joint count (TJC) ≥3 (of 68), a swollen joint count (SJC) ≥3 (of 66), and ≥1 active psoriatic lesion and/or a documented history of psoriasis.

In BE OPTIMAL, patients with current or prior exposure to any biologics for the treatment of PsA or psoriasis were excluded. In BE COMPLETE, patients were required to have had a prior inadequate response or intolerance to one or two TNFi for either PsA or psoriasis; patients with current or previous exposure to any other biologics were excluded.

### MTX exposure

MTX was permitted in both BE OPTIMAL and BE COMPLETE up to a maximum dose of 25 mg/week or the maximum tolerated dose, whichever was lower. Patients were required to have initiated MTX at least 12 weeks before Week 0 (baseline), with a stable dose for at least 8 weeks before randomization. The study protocols specified that dose, dosing schedule, and administration route (oral or subcutaneous) should remain stable until Week 16 and strongly recommended taking folic acid supplementation with MTX. Rescue therapy using MTX was permitted; after Week 16 of both studies, MTX could be added or increased to the lowest dose of 25 mg/week or the maximum tolerated dose. Administration route could be changed from subcutaneous to oral or vice versa.

### Assessments and outcomes

Time points for efficacy and safety assessments were reported previously.^15^, ^16^, ^17^ Outcomes are presented by randomization group (bimekizumab or placebo) and by concomitant MTX treatment at baseline for BE OPTIMAL and BE COMPLETE.

Efficacy outcomes reported include improvements from baseline of ≥20%, ≥50%, and ≥70% in the American College of Rheumatology (ACR20/50/70) response criteria,^19^ improvements from baseline of ≥75%, ≥90%, and 100% in the Psoriasis Area and Severity Index criteria (PASI75/90/100),^20^ minimal and very low disease activity (MDA, VLDA; achievement of ≥5/7 or 7/7, respectively, of the following criteria: TJC ≤1, SJC ≤1, either PASI ≤1 or body surface area [BSA] ≤3%, patients’ pain visual analog scale [VAS] ≤15, Patient's Global Assessment of PsA [PGA‐PsA] ≤20 [VAS], Health Assessment Questionnaire‐Disability Index [HAQ‐DI] ≤0.5,^21^ and tender entheseal points ≤1 [measured with the Leeds Enthesitis Index (LEI)]),^22^ ACR50+PASI100, resolution of enthesitis (LEI=0), resolution of dactylitis (Leeds Dactylitis Index [LDI]=0),^23^ resolution of nail psoriasis (modified Nail Psoriasis Severity Index [mNAPSI]=0), HAQ‐DI score, and Disease Activity Index for Psoriatic Arthritis (DAPSA) remission (REM) and low disease activity (LDA).^24^

Safety outcomes, reported to Week 52, include treatment‐emergent adverse events (TEAEs), serious TEAEs, and study discontinuations due to TEAEs. Other safety results reported include drug‐related TEAEs, severe TEAEs, deaths, and other safety topics of interest (uveitis, adjudicated major adverse cardiovascular events [MACE], neutropenia, infections, fungal infections, hypersensitivity, injection‐site reactions, adjudicated suicidal ideation and behavior, liver function test changes and/or enzyme elevations, adjudicated inflammatory bowel disease [IBD], and malignancies).

### Statistical analysis

Post hoc analyses of responses and changes from baseline were conducted relative to baseline values (Week 0) of BE OPTIMAL and BE COMPLETE. Data are reported for the randomized sets, side‐by‐side for each study, by treatment arm, and by concomitant MTX treatment at baseline. Because this analysis was post hoc, no statistical comparisons were made. We included 95% confidence intervals (CIs) for efficacy data in Tables and Figures, where available.    

Missing data were imputed using non‐responder imputation for dichotomous outcomes and multiple imputation for continuous outcomes. Worst‐category imputation was used for DAPSA, in which missing data were considered in the worst category. Patients who did not enroll in BE VITAL were included in the analysis of BE COMPLETE data to Week 16 and were considered non‐responders for response variables and were imputed using multiple imputation for continuous variables thereafter. Safety data are reported for all randomized patients who received one or more doses of bimekizumab. Exposure‐adjusted incidence rates (EAIRs) per 100 patient‐years (PY) are reported for safety events, when available.

## RESULTS

### Patient disposition and baseline characteristics

Patient demographics and baseline characteristics for patients randomized to bimekizumab or placebo in BE OPTIMAL and BE COMPLETE are shown in Table 1. Patient disposition is provided in Supplementary Figure S1. Of the patients randomized to bimekizumab or placebo in BE OPTIMAL (n = 712), 415 (58.3%) patients were receiving MTX (+MTX) and 297 (41.7%) were not (−MTX); 645 (90.6%) completed Week 52 (383 +MTX [92.3%], 262 −MTX [88.2%]), including 7 patients who completed Week 52 while not receiving randomized treatment (4 +MTX; 3 −MTX). In BE COMPLETE, there were 170 (42.5%) +MTX patients and 230 (57.5%) −MTX patients; of all patients, 388 (97.0%) completed Week 16, 377 (94.3%) entered the BE VITAL OLE (165 +MTX [97.1%], 212 −MTX [92.2%]), and 347 (86.8%) completed Week 52 (156 +MTX [91.8%], 191 −MTX [83.0%]), including 4 patients not receiving randomized treatment (3 +MTX; 1 −MTX).

**Table 1:** Patient demographics and baseline characteristics*

	BE OPTIMAL (bDMARD‐naïve)				BE COMPLETE (TNFi‐IR)			
	Placebo n = 281		BKZ 160 mg Q4W n = 431		Placebo n = 133		BKZ 160 mg Q4W n = 267	
	+MTX, n = 163	−MTX, n = 118	+MTX, n = 252	−MTX, n = 179	+MTX, n = 51	−MTX, n = 82	+MTX, n = 119	−MTX, n = 148
Age, mean (SD), y	48.2 (11.5)	49.3 (12.1)	47.8 (12.6)	49.6 (12.4)	50.2 (13.4)	52.0 (12.6)	50.9 (13.3)	49.5 (11.6)
Male, n (%)	72 (44.2)	55 (46.6)	122 (48.4)	79 (44.1)	23 (45.1)	37 (45.1)	63 (52.9)	67 (45.3)
BMI, mean (SD)	29.4 (6.1)	29.9 (6.0)	29.1 (6.5)	29.4 (7.2)	27.7 (4.5)	29.9 (5.8)	29.8 (6.3)	30.4 (6.6)
Time since first diagnosis of PsA, mean (SD), y	5.4 (6.2)	6.0 (7.0)^a^	5.8 (7.3)^a^	6.2 (7.3)^b^	8.7 (7.4)	9.6 (8.6)^c^	9.4 (9.7)	9.8 (10.0)^c^
Duration taking MTX,^d^ mean (SD), y	2.3 (2.9)	N/A	2.5 (2.9)	N/A	3.0 (4.0)	N/A	2.9 (2.9)	N/A
Weekly dose of MTX at baseline, mean (SD), mg	17.1 (5.1)^e^	N/A	16.7 (5.3)^f^	N/A	19.0 (5.2)	N/A	16.9 (5.8)	N/A
TJC (of 68 joints), mean (SD)	16.4 (12.3)	18.0 (12.7)	16.6 (11.8)	17.1 (11.8)	20.7 (14.4)	18.4 (14.2)	16.7 (10.4)	19.8 (15.6)
SJC (of 66 joints), mean (SD)	10.0 (7.8)	8.8 (6.5)	9.1 (6.4)	8.8 (5.9)	12.6 (9.3)	8.8 (7.0)	9.3 (6.5)	10.0 (8.3)
hs‐CRP, mean (SD), mg/L	12.3 (26.5)	10.1 (14.6)	9.1 (14.0)	8.2 (10.9)	12.8 (20.1)	10.9 (17.5)	14.8 (21.7)	10.4 (13.7)
Psoriasis with ≥3% BSA, n (%)	83 (50.9)	57 (48.3)	126 (50.0)	91 (50.8)	35 (68.6)	53 (64.6)	80 (67.2)	96 (64.9)
PASI score,^g^ mean (SD)	7.6 (5.3)	8.4 (6.1)	7.7 (6.4)	8.8 (7.4)	8.2 (7.2)	8.6 (6.2)	10.4 (9.7)	10.0 (8.6)
Presence of enthesitis (LEI >0), n (%)	36 (22.1)	34 (28.8)	82 (32.5)^f^	61 (34.1)^c^	15 (29.4)	21 (25.6)^c^	48 (40.3)	58 (39.2)
Score,^h^ mean (SD)	2.8 (1.6)	3.0 (1.5)	2.4 (1.4)^f^	2.6 (1.5)^c^	3.0 (1.6)	2.9 (1.6)	2.6 (1.5)	2.6 (1.5)
Presence of dactylitis (LDI >0), n (%)	22 (13.5)	11 (9.3)	28 (11.1)^b^	28 (15.6)^c^	9 (17.6)	5 (6.1)^c^	7 (5.9)	27 (18.2)
Score,^i^ mean (SD)	46.1 (36.6)	49.9 (50.6)	38.2 (32.0)^b^	55.3 (69.6)^c^	82.3 (159.0)	38.0 (28.8)	42.0 (37.3)	80.6 (126.3)
Presence of nail psoriasis (mNAPSI >0), n (%)	92 (56.4)	64 (54.2)	146 (57.9)^b^	98 (54.7)^c^	38 (74.5)	45 (54.9)^c^	72 (60.5)	87 (58.8)
Score,^j^ mean (SD)	4.1 (2.2)	3.8 (2.0)	4.0 (2.4)^b^	4.2 (2.5)^c^	5.3 (3.0)	3.8 (2.5)	4.5 (3.0)	4.2 (2.5)
HAQ‐DI score, mean (SD)	0.90 (0.60)	0.88 (0.62)	0.78 (0.59)^c^	0.87 (0.58)	1.09 (0.69)	1.00 (0.69)	1.01 (0.57)	0.94 (0.60)
Patient's assessment of pain VAS score, mean (SD)	57.3 (23.4)	56.2 (23.2)	53.1 (24.3)^c^	54.4 (24.3)	64.3 (24.2)	60.1 (24.8)	59.0 (23.3)	57.8 (25.0)
Physician's global assessment score, mean (SD)	58.1 (15.3)	56.1 (14.7)^c^	56.6 (16.0)^k^	58.1 (16.8)^c^	58.9 (17.2)	56.9 (19.8)^c^	61.2 (15.9)	57.7 (18.1)
Patient's global assessment score, mean (SD)	60.1 (23.7)	56.5 (23.1)	53.1 (23.5)^c^	56.3 (23.3)	64.6 (22.3)	62.0 (21.9)	59.1 (22.6)	61.7 (22.4)

* Randomized set. bDMARD, biologic disease‐modifying antirheumatic drug; BKZ, bimekizumab; BMI, body mass index; BSA, body surface area; HAQ‐DI, Health Assessment Questionnaire ‐ Disability Index; hs‐CRP, high‐sensitivity C‐reactive protein; LDI, Leeds Dactylitis Index; LEI, Leeds Enthesitis Index; mNAPSI, modified Nail Psoriasis Severity Index; +MTX, with methotrexate; −MTX, without methotrexate; PASI, Psoriasis Area and Severity Index; PsA, psoriatic arthritis; Q4W, every four weeks; SJC, swollen joint count; TJC, tender joint count; TNFi‐IR, prior inadequate response or intolerance to tumor necrosis factor inhibitors; VAS, visual analog scale.

^a^ Data missing for two patients.

^b^ Data missing for six patients.

^c^ Data missing for one patient.

^d^ Before first administration of study drug.

^e^ Data missing for three patients.

^f^ Data missing for five patients.

^g^ In patients with ≥3% BSA affected by psoriasis at baseline.

^h^ In patients with an LEI score >0 at baseline.

^i^ In patients with an LDI score >0 at baseline.

^j^ In patients with an mNAPSI score >0 at baseline.

^k^ Data missing for four patients.

Patient demographics and baseline disease characteristics were generally similar for +/−MTX patient subgroups across treatment arms within each study, with the exception of slightly elevated high‐sensitivity C‐reactive protein levels and a slightly higher SJC in +MTX versus −MTX patients (Table 1). The overall mean (SD) weekly dose of MTX at baseline received by patients randomized to placebo or bimekizumab ranged from 16.7 (5.3) to 19.0 (5.2) mg. Baseline characteristics and disposition for patients receiving the reference drug (adalimumab) +/−MTX treatment are reported in Supplementary Table S1 and Supplementary Figure S1.

### Efficacy

In both BE OPTIMAL (bDMARD‐naïve) and BE COMPLETE (TNFi‐IR) studies, improvements across all measured PsA domains, including joints and skin, were observed with bimekizumab treatment at Week 16 and maintained through 52 weeks in both the +MTX and −MTX groups.

**Figure 1:**
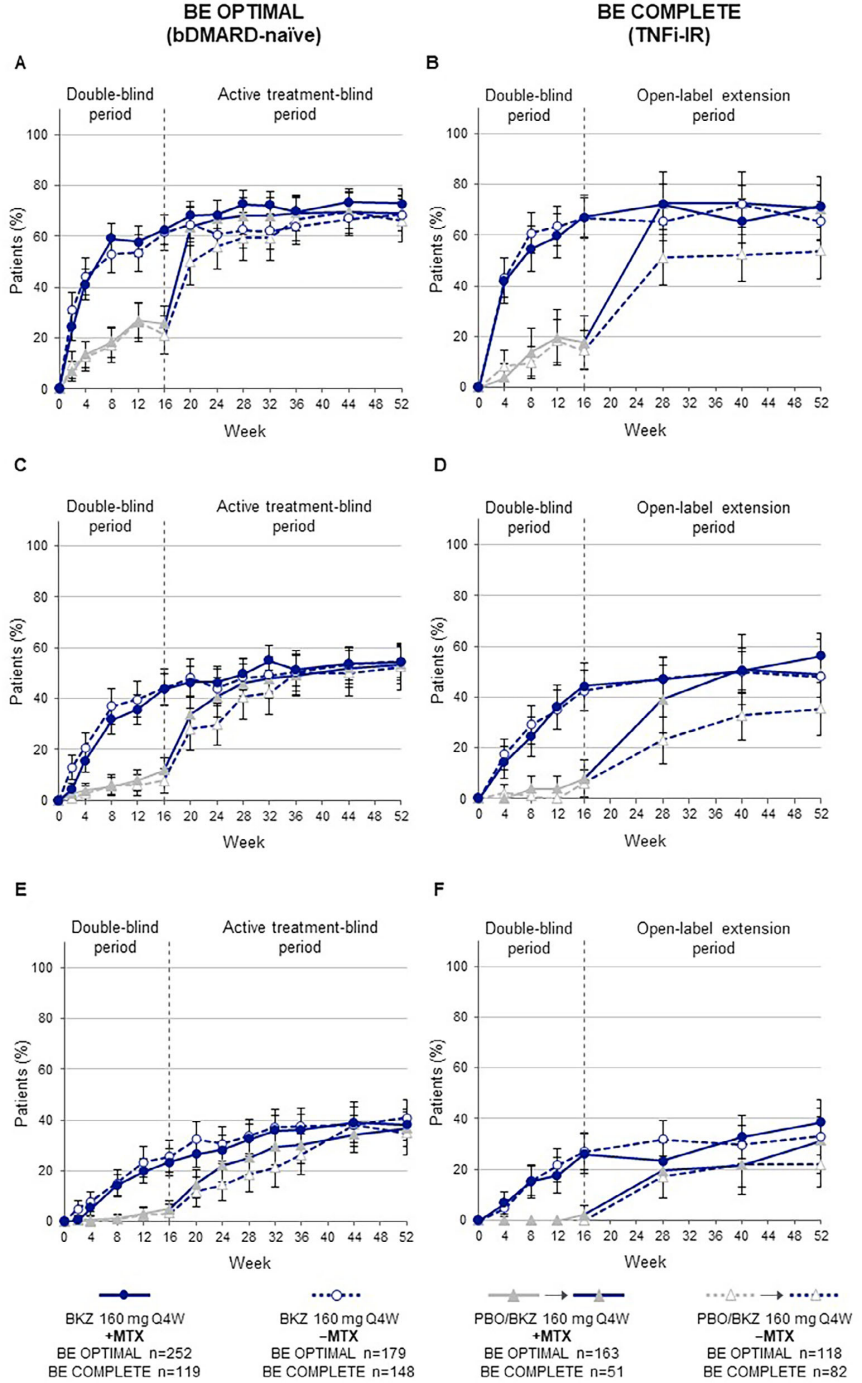
(A and B) ACR20, (C and D) ACR50, and (E and F) ACR70 responses (95% CI) to Week 52 by baseline MTX treatment (NRI). Randomized set. Error bars show 95% CIs. A 95% CI could not be evaluated for patients randomized to PBO at Week 4 in ACR70. ACR20/50/70, ≥20%/50%/70% improvement in American College of Rheumatology response criteria; bDMARD, biologic disease‐modifying antirheumatic drug; BKZ, bimekizumab; CI, confidence interval; +MTX, with methotrexate; −MTX, without methotrexate; NRI, non‐responder imputation; PBO, placebo; Q4W, every four weeks; TNFi‐IR, prior inadequate response or intolerance to tumor necrosis factor inhibitors.

The proportion of patients who received bimekizumab treatment from baseline achieving an ACR50 response was sustained from Week 16 to Week 52 for both +MTX and −MTX patients in BE OPTIMAL (+MTX: 54.4%; −MTX: 54.7%) and BE COMPLETE (+MTX: 56.3%; −MTX: 48.0%; Figure 1). Both +MTX and −MTX patients who switched from placebo to bimekizumab at Week 16 demonstrated improvements in ACR50 response to Week 52 of BE OPTIMAL (+MTX: 53.4%; −MTX: 52.5%) and BE COMPLETE (+MTX: 49.0%; −MTX: 35.4%; Figure 1). Similar trends within treatment arms were observed across additional joint thresholds (ACR20/70; Figure 1).

**Figure 2:**
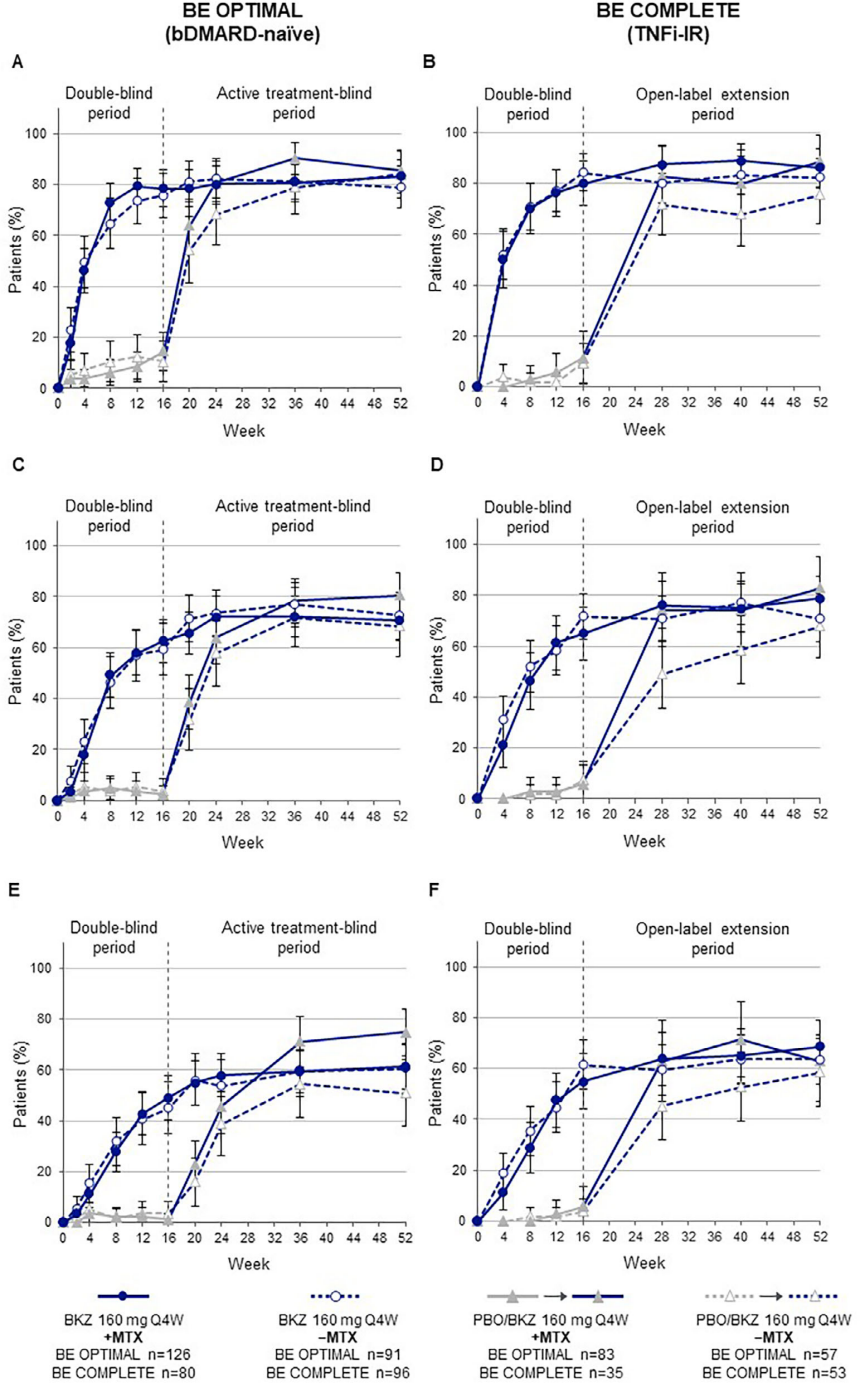
(A and B) PASI75, (C and D) PASI90, and (E and F) PASI100 responses (95% CI) to Week 52 by baseline MTX treatment (NRI). Randomized set, in patients with ≥3% BSA affected by psoriasis at baseline. Error bars show 95% CIs. A 95% CI could not be evaluated for patients randomized to PBO at week 2 in PASI100. bDMARD, biologic disease‐modifying antirheumatic drug; BKZ, bimekizumab; BSA, body surface area; CI, confidence interval; +MTX, with methotrexate; −MTX, without methotrexate; NRI, non‐responder imputation; PASI75/90/100, ≥75%/90%/100% improvement in Psoriasis Area and Severity Index criteria; PBO, placebo; Q4W, every four weeks; TNFi‐IR, previous inadequate response or intolerance to tumor necrosis factor inhibitors.

Among patients with psoriasis affecting ≥3% BSA, the proportion of bimekizumab‐randomized patients achieving complete skin clearance (PASI100) was sustained from Week 16, and for both +MTX and −MTX patients in BE OPTIMAL (+MTX: 61.1%; −MTX: 60.4%) and BE COMPLETE (+MTX: 68.8%; −MTX: 63.5%; Figure 2). The proportions of patients with PASI100 after switching from placebo to bimekizumab at week 16 increased markedly to Week 52 for both +MTX and −MTX patients in BE OPTIMAL (+MTX: 74.7%; −MTX: 50.9%) and BE COMPLETE (+MTX: 62.9%; −MTX: 58.5%; Figure 2). Similar trends within treatment arms were observed across additional skin thresholds (PASI75/90; Figure 2).

**Figure 3:**
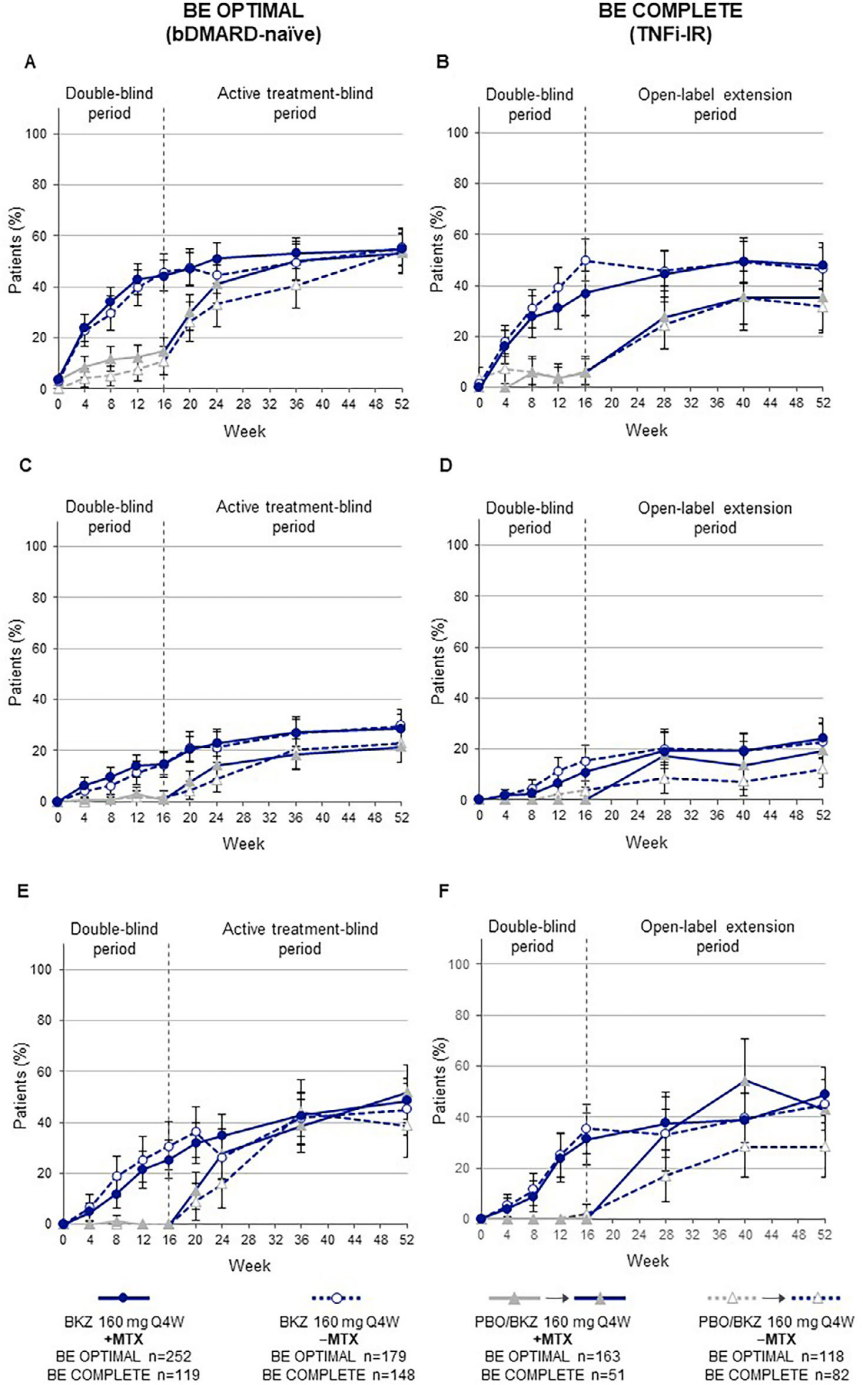
Additional composite efficacy outcomes (95% CI) to Week 52 by baseline MTX treatment (NRI). (A and B) MDA. (C and D) VLDA. (E and F) ACR50+PASI100 (in patients with psoriasis, BSA≥ 3% at baseline). Randomized set. Error bars show 95% CIs. BE OPTIMAL: BKZ and +MTX n = 126, BKZ and −MTX n = 91, PBO/BKZ and +MTX n = 83, PBO/BKZ and −MTX n = 57; BE COMPLETE: BKZ and +MTX n = 80, BKZ and −MTX n = 96, PBO/BKZ and +MTX n = 35, PBO/BKZ and −MTX n = 53. ACR50+PASI100, achievement of both ≥50% improvement in American College of Rheumatology response criteria and 100% improvement in Psoriasis Area and Severity Index criteria; bDMARD, biologic disease‐modifying antirheumatic drug; BKZ, bimekizumab; BSA, body surface area; CI, confidence interval; MDA, minimal disease activity; +MTX, with methotrexate; −MTX, without methotrexate; NRI, non‐responder imputation; PBO, placebo; Q4W, every four weeks; TNFi‐IR, prior inadequate response or intolerance to tumor necrosis factor inhibitors; VLDA, very low disease activity.

The proportion of patients who received bimekizumab from baseline achieving an MDA response was similarly sustained from Week 16 to Week 52 for both +MTX and −MTX patients in BE OPTIMAL (+MTX: 54.8%; −MTX: 55.3%) and BE COMPLETE (+MTX: 47.9%; −MTX: 46.6%; Figure 3). Patients who switched from placebo to bimekizumab at Week 16 demonstrated improvements to Week 52 for both +MTX and −MTX patients in BE OPTIMAL (+MTX: 53.4%; −MTX: 54.2%) and BE COMPLETE (+MTX: 35.3%; −MTX: 31.7%; Figure 3). Similar trends within treatment arms were observed when using other composite measures of efficacy (VLDA, ACR50+PASI100; Figure 3).

Results for resolution of enthesitis, dactylitis, and nail psoriasis HAQ‐DI change from baseline; and proportions of patients in DAPSA LDA plus REM and REM states were generally similar between trials and treatment arms and are reported in Table 2. Efficacy data for the reference arm from BE OPTIMAL are reported in Supplementary Table S2 and Supplementary Figures S2–S4. Observed case data are reported in Supplementary Figures S5–S7.

**Table 2:** Additional efficacy endpoints (95% CI) at Week 52 by baseline MTX treatment (NRI, MI, WCI)*

	BE OPTIMAL (bDMARD‐naïve)				BE COMPLETE (TNFi‐IR)			
	Placebo (n = 281)		BKZ 160 mg Q4W (n = 431)		Placebo (n = 133)		BKZ 160 mg Q4W (n = 267)	
	+MTX, n = 163	−MTX, n = 118	+MTX, n = 252	−MTX, n = 179	+MTX, n = 51	−MTX, n = 82	+MTX, n = 119	−MTX, n = 148
Enthesitis resolution,^a^ n/N (%)	24/36 (66.7)	20/34 (58.8)	53/82 (64.6)	34/61 (55.7)	9/15 (60.0)	12/21 (57.1)	26/48 (54.2)	34/58 (58.6)
95% CI	51.3 to 82.1	42.3 to 75.4	54.3 to 75.0	43.3 to 68.2	35.2 to 84.8	36.0 to 78.3	40.1 to 68.3	45.9 to 71.3
Dactylitis resolution,^b^ n/N (%)	18/22 (81.8)	11/11 (100)	21/28 (75.0)	24/28 (85.7)	8/9 (88.9)	4/5 (80.0)	7/7 (100)	22/27 (81.5)
95% CI	65.7 to 97.9	NE	59.0 to 91.0	72.8 to 98.7	68.4 to 100	44.9 to 100	100 to 100	66.8 to 96.1
Nail psoriasis resolution,^c^ n/N (%)	68/92 (73.9)	43/64 (67.2)	100/146 (68.5)	60/98 (61.2)	25/38 (65.8)	26/45 (57.8)	50/72 (69.4)	57/87 (65.5)
95% CI	64.9 to 82.9	55.7 to 78.7	61.0 to 76.0	51.6 to 70.9	50.7 to 80.9	43.3 to 72.2	58.8 to 80.1	55.5 to 75.5
HAQ‐DI CfB (MI), mean (SE)	−0.37 (0.04)	−0.37 (0.05)	−0.30 (0.03)	−0.38 (0.04)	−0.43 (0.09)	−0.29 (0.07)	−0.40 (0.04)	−0.38 (0.04)
95% CI	−0.45 to −0.29	−0.47 to −0.28	−0.36 to −0.24	−0.46 to −0.30	−0.60 to −0.25	−0.43 to −0.16	−0.49 to −0.32	−0.46 to −0.29
DAPSA (WCI)								
LDA and REM,^d^ n (%)	91 (55.8)	64 (54.2)	149 (59.1)	97 (54.2)	22 (43.1)	32 (39.0)	58 (48.7)	76 (51.4)
REM, n (%)	30 (18.4)	16 (13.6)	57 (22.6)	43 (24.0)	7 (13.7)	5 (6.1)	18 (15.1)	25 (16.9)
95% CI	12.0 to 27.1	7.5 to 23.3	16.7 to 29.8	17.0 to 32.8	5.7 to 29.7	2.1 to 16.3	8.7 to 25.1	10.6 to 25.9

* Randomized set. NRI unless otherwise stated. bDMARD, biologic disease‐modifying antirheumatic drug; BKZ, bimekizumab; CfB, change from baseline; CI, confidence interval; DAPSA, Disease Activity Index for Psoriatic Arthritis; HAQ‐DI, Health Assessment Questionnaire ‐ Disability Index; LDA, low disease activity; LDI, Leeds Dactylitis Index; LEI, Leeds Enthesitis Index; MI, multiple imputation; mNAPSI, modified Nail Psoriasis Severity Index; +MTX, with methotrexate; −MTX, without methotrexate; NE, not evaluable; NRI, non‐responder imputation; Q4W, every four weeks; REM, remission; TNFi‐IR, prior inadequate response or intolerance to tumor necrosis factor inhibitors; WCI, worst‐category imputation.

^a^ In patients with an LEI score >0 at baseline.

^b^ In patients with an LDI score >0 at baseline.

^c^ In patients with an mNAPSI score >0 at baseline.

^d^ Calculated by hand; 95% CIs are not available.

### Safety

Up to 52 weeks, patients receiving ≥1 dose bimekizumab had similar overall EAIR/100 PY of TEAEs for both +MTX and −MTX patients in both BE OPTIMAL and BE COMPLETE (Table 3). Safety data for the reference arm from BE OPTIMAL are reported in Supplementary Table S3. In BE OPTIMAL, 325 of 410 (79.3%; EAIR/100 PY: 219.3) +MTX patients reported ≥1 TEAE, as did 230 of 292 (78.8%; EAIR/100 PY: 227.6) −MTX patients. In BE COMPLETE, ≥1 TEAE was reported by 105 of 168 (62.5%; EAIR/100 PY: 118.2) +MTX patients and 138 of 220 (62.7%; EAIR/100 PY: 132.6) −MTX patients.

**Table 3:** Safety outcomes to Week 52*

	BE OPTIMAL (bDMARD‐naïve)		BE COMPLETE (TNFi‐IR)	
	BKZ 160 mg Q4W (n = 702)^a^		BKZ 160 mg Q4W (n = 388)^a^	
n (%) [EAIR/100 PY]^b^	+MTX, n = 410 (PYs: 355.4)	−MTX, n = 292 (PYs: 247.2)	+MTX, n = 168 (PYs: 149.4)	−MTX, n = 220 (PYs: 190.4)
Any TEAE	325 (79.3) [219.3]	230 (78.8) [227.6]	105 (62.5) [118.2]	138 (62.7) [132.6]
Serious TEAEs	26 (6.3) [7.5]	20 (6.8) [8.4]	9 (5.4) [6.2]	14 (6.4) [7.6]
Study discontinuation due to TEAE	10 (2.4) [2.8]	11 (3.8) [4.5]	3 (1.8) [2.0]	13 (5.9) [7.0]
Drug‐related TEAEs^c^	133 (32.4)	91 (31.2)	34 (20.2)	53 (24.1)
Severe TEAEs	13 (3.2)	10 (3.4)	6 (3.6)	11 (5.0)
Deaths^d^	1 (0.2)	0	1 (0.6)	0
Most frequent TEAEs^e^				
Nasopharyngitis	41 (10.0) [12.5]	43 (14.7) [19.4]	9 (5.4) [6.3]	14 (6.4) [7.6]
Upper respiratory tract infection	34 (8.3) [10.2]	16 (5.5) [6.7]	8 (4.8) [5.5]	4 (1.8) [2.1]
Urinary tract infection	30 (7.3) [8.7]	13 (4.5) [5.4]	7 (4.2) [4.8]	16 (7.3) [8.8]
Headache	20 (4.9) [5.9]	21 (7.2) [9.0]	4 (2.4) [2.7]	6 (2.7) [3.2]
Oral candidiasis^f^	23 (5.6) [6.7]	15 (5.1) [6.2]	12 (7.1) [8.4]	12 (5.5) [6.5]
Diarrhea	20 (4.9) [5.8]	16 (5.5) [6.7]	3 (1.8) [2.0]	5 (2.3) [2.7]
Pharyngitis	21 (5.1) [6.1]	11 (3.8) [4.6]	1 (0.6) [0.7]	1 (0.5) [0.5]
SARS‐CoV‐2 (COVID‐19)	10 (2.4) [2.8]	10 (3.4) [4.1]	11 (6.5) [7.6]	17 (7.7) [9.2]
Uveitis	0	0	0	0
Adjudicated MACE^g^	3 (0.7) [0.9]	1 (0.3) [0.4]	1 (0.6) [0.7]	1 (0.5) [0.5]
Neutropenia^h^	8 (2.0) [2.3]	3 (1.0) [1.2]	2 (1.2) [1.4]	3 (1.4) [1.6]
Infections				
Serious	3 (0.7) [0.9]	3 (1.0) [1.2]	3 (1.8) [2.0]	4 (1.8) [2.1]
Opportunistic	3 (0.7) [0.9]	6 (2.1) [2.5]	1 (0.6) [0.7]	0
Hypersensitivity^i^	30 (7.3) [8.8]	29 (9.9) [12.4]	5 (3.0) [3.4]	14 (6.4) [7.7]
Dermatitis and eczema	9 (2.2) [2.6]	15 (5.1) [6.3]	3 (1.8) [2.0]	5 (2.3) [2.7]
Injection‐site reactions	9 (2.2) [2.6]	6 (2.1) [2.5]	1 (0.6) [0.7]	5 (2.3) [2.7]
Adjudicated suicidal ideation and behavior	0	0	0	0
Liver function test changes/enzyme elevations				
ALT >3 × ULN	11/410 (2.7)	4/291 (1.4)	5/168 (3.0)	3/220 (1.4)
AST or ALT >3 × ULN	16/410 (3.9)	8/291 (2.7)	7/168 (4.2)	5/220 (2.3)
Adjudicated IBD^j^	2 (0.5) [0.6]	2 (0.7) [0.8]	0	0
Malignancies excluding nonmelanoma skin cancer				
Colon cancer	1 (0.2) [0.3]	0	0	0
Chronic lymphocytic leukemia stage 0	0	1 (0.3) [0.4]	0	0
Endometrial cancer stage 1	0	0	0	1 (0.5) [0.5]
Papillary thyroid cancer	0	1 (0.3) [0.4]	0	0
Prostate cancer	0	0	0	1 (0.5) [0.5]
Recurrent gastric cancer	0	0	1 (0.6) [0.7]	0
Nonmelanoma skin cancer				
Squamous cell carcinoma	1 (0.2) [0.3]	0	0	0
Basal cell carcinoma	1 (0.2) [0.3]	2 (0.7) [0.8]	0	0

* Safety set. ALT, alanine aminotransferase; AST, aspartate aminotransferase; bDMARD, biologic disease‐modifying antirheumatic drug; BKZ, bimekizumab; EAIR, exposure‐adjusted incidence rate; IBD, inflammatory bowel disease; MACE, major adverse cardiovascular event; +MTX, with methotrexate; −MTX, without methotrexate; PY, patient‐year; Q4W, every four weeks; TEAE, treatment‐emergent adverse event; TNFi‐IR, prior inadequate response or intolerance to tumor necrosis factor inhibitors; ULN, upper limit of normal.

^a^ Includes placebo/BKZ‐treated patients (events after switch only).

^b^ Reported where available.

^c^ Assessed by reporter.

^d^ BE OPTIMAL: motorcycle accident, unrelated to treatment. BE COMPLETE: sudden death, no autopsy conducted.

^e^ Events occurring in ≥5% of BKZ‐treated patients in either study.

^f^ All infections were mild or moderate in severity; none were serious. One −MTX BKZ‐treated patient discontinued in BE OPTIMAL.

^g^ BE OPTIMAL +MTX: one case each of myocardial infarction, ischemic stroke, and thrombotic cerebral infarction. The case of ischemic stroke was deemed by the investigator to be related to study medication; −MTX: one case of cerebrovascular accident. BE COMPLETE +MTX: one case of sudden death and one case of cerebral hemorrhage, both unrelated to treatment.

^h^ BE OPTIMAL +MTX: seven neutropenia cases, one case of decreased neutrophil count; −MTX: three neutropenia cases. BE COMPLETE +MTX: two neutropenia cases; −MTX: two neutropenia cases and one case of decreased neutrophil count.

^i^ No cases were serious.

^j^ Including definite or probable TEAEs.

The majority of TEAEs were mild or moderate in severity across both studies; there were similar rates of serious and drug‐related adverse events in +MTX and −MTX subgroups. Incidence rates of study discontinuation due to TEAEs were numerically lower for +MTX patients than for −MTX patients. Incidence rates of elevated liver enzymes (three times the upper limit of normal) were numerically higher for +MTX patients than for −MTX patients.

*Candida* infections were reported in 7.1% (29, EAIR/100 PY: 8.5) of +MTX patients and 8.6% (25, EAIR/100 PY: 10.6) of −MTX patients to Week 52 of BE OPTIMAL. In BE COMPLETE, 7.1% (12, EAIR/100 PY: 8.4) of +MTX patients and 5.9% (13, EAIR/100 PY: 7.1) of −MTX patients had a *Candida* infection. Similar proportions of patients reported fungal infections not elsewhere classified up to Week 52 (BE OPTIMAL +MTX 4.6% [19, EAIR/100 PY: 5.5], −MTX 3.4% [10, EAIR/100 PY: 4.1]; BE COMPLETE +MTX 2.4% [4, EAIR/100 PY: 2.7], −MTX 3.6% [8, EAIR/100 PY: 4.3]).

There were two deaths up to Week 52, both in patients with baseline MTX treatment: one death due to a motorcycle accident in BE OPTIMAL and one sudden death in a patient with a history of cardiac events in BE COMPLETE. Neither event was reported to be related to study medication.

## DISCUSSION

Treatment with bimekizumab was demonstrated to deliver consistent and sustained clinical efficacy to 52 weeks in bDMARD‐naïve and TNFi‐IR patients with active PsA, irrespective of concomitant MTX treatment. Bimekizumab demonstrated efficacy in joints, skin, and composite outcomes, with similar results across studies, indicating effectiveness irrespective of patients’ prior biologic treatment. Similar results across these patient populations are of clinical interest because TNFi‐IR patients are typically more difficult to treat, cycle through therapies with varying mechanisms of action more quickly than patients who are tolerant to TNFi, and have reduced response rates overall.^25^, ^26^ The efficacy observed in these studies in the skin domain was consistent in both +MTX and −MTX patients, as well as in bDMARD‐naïve and TNFi‐IR patients.

The similar efficacy, irrespective of concomitant MTX, across outcomes in both bDMARD‐naïve and TNFi‐IR populations may result from the distinctive contribution made by dual inhibition of IL‐17A and IL‐17F with bimekizumab. This unique mechanism of action was effective irrespective of prior biologic treatment, and was not impacted by concomitant MTX treatment because the mechanism of action of MTX has no overlap with that of bimekizumab.^27^, ^28^ These results may be of use to clinicians when choosing appropriate treatments with patients because they suggest that MTX may not be required for maintenance of response with bimekizumab. Future studies are required to evaluate the distinctive role of IL‐17F in terms of mechanisms of therapeutic resistance over time.

The overall safety profile of bimekizumab in BE OPTIMAL and BE COMPLETE and across both the +MTX and −MTX subgroups was similar between studies and in line with previously reported results.^16^, ^17^, ^29^ Rates of fungal infections were consistent between studies, and the incidence of fungal infections was unaffected by concomitant MTX treatment. For safety outcomes such as liver enzyme level elevations (three times the upper limit of normal), numerically higher values were observed in the +MTX patients in both trials compared with the −MTX group, which is in line with reported increases in hepatic events in the literature^7^, ^30^, ^31^, ^32^; however, these studies were not able to evaluate significance, and thus the results should be interpreted with caution. As concomitant MTX is not required for bimekizumab efficacy, patients may benefit from a reduced burden with respect to fewer medications and the reduction of MTX‐associated issues, such as hepatic events, as well as the ongoing monitoring requirements.^30^, ^32^

The results presented here contribute to the understanding of the impact of concomitant MTX on the efficacy and safety of biologics. This is of particular importance given the emphasis on safety in current treatment guidelines^3^, ^4^, ^5^, ^33^ and the adverse events and tolerability concerns associated with MTX, which can result in discontinuation.^34^, ^35^ Additional considerations include the unclear effect on male fertility^36^, ^37^ and the general metabolic profile of patients with PsA, which can put them at a higher risk for adverse events, such as hepatic events requiring frequent monitoring.^7^, ^30^, ^31^, ^32^, ^35^ By improving the understanding of bDMARD treatment +/−MTX, shared treatment decisions can be made with the aim of controlling symptoms, reducing adverse events, and improving patient quality of life. Based on the efficacy and safety results presented here, the addition of MTX may not be required with bimekizumab to treat bDMARD‐naïve or TNFi‐IR patients with PsA. In general, patients treated with MTX did not experience higher efficacy in the outcomes reported here, nor was a different safety profile observed between +MTX and −MTX patients.

Strengths of this analysis include the alignment of recruitment and study centers across the phase 3 trial program for bimekizumab in PsA, which lends itself to concurrent analyses of the aforementioned patient populations; this post hoc analysis assesses efficacy in two groups of +MTX and −MTX patients. As approximately half of the patients in both studies were receiving MTX, the sample sizes for both subgroups were generally large for the majority of outcomes. Additionally, the assessment of the 52‐week BE OPTIMAL trial and after 52 weeks of treatment for patients in BE COMPLETE/BE VITAL OLE evaluated bimekizumab over a substantial treatment duration. Finally, both studies reported a low dropout rate, indicating a high level of tolerability overall and in both +MTX and −MTX subgroups.

A limitation of the present study is the post hoc nature of the analysis; thus, this study was not powered to evaluate the benefit of MTX in combination with bimekizumab. The 95% CIs are included for efficacy data, allowing for an understanding of what the result of a comparison could be. Safety results are reported using descriptive analysis from the trials, and therefore significant differences in safety events could not be detected between treatment arms or between MTX groups. Additionally, the sample size of the placebo treatment group in BE COMPLETE was smaller when further stratified by MTX for some end points, such as those assessing enthesitis and dactylitis. As eligible patients could enroll in the OLE BE VITAL study after Week 52 from BE OPTIMAL and Week 16 from BE COMPLETE, future publications will report 2‐year data from BE VITAL for longer‐term analysis.

In conclusion, treatment with bimekizumab demonstrated consistent, sustained clinical efficacy to 52 weeks in bDMARD‐naïve and TNFi‐IR patients with active PsA, irrespective of concomitant MTX administration. Results were similar between the BE OPTIMAL and BE COMPLETE study populations, and bimekizumab was well tolerated in patients with PsA in both +MTX and −MTX groups.

## AUTHOR CONTRIBUTIONS

All authors contributed to at least one of the following manuscript preparation roles: conceptualization AND/OR methodology, software, investigation, formal analysis, data curation, visualization, and validation AND drafting or reviewing/editing the final draft. As corresponding author, Professor McInnes confirms that all authors have provided the final approval of the version to be published, and takes responsibility for the affirmations regarding article submission (eg, not under consideration by another journal), the integrity of the data presented, and the statements regarding compliance with institutional review board/Helsinki Declaration requirements.

## ROLE OF THE STUDY SPONSOR

Support for third‐party medical writing and editorial assistance for this article, provided by Laura Mawdsley, MSc, Costello Medical, Cambridge, UK, was funded by UCB Pharma in accordance with Good Publication Practice guidelines (http://www.ismpp.org/gpp3). UCB Pharma had a role in the study design, collection, analysis and interpretation of the data, manuscript review and the decision to submit the manuscript for publication. Publication of this article was contingent upon approval by UCB Pharma.

## Supporting information


Disclosure form

**Data S1:** CONSORT 2010 checklist of information to include when reporting a randomised trial

**Supplementary Figure S1:** CONSORT diagrams for (A) BE OPTIMAL and (B) BE COMPLETE
**Supplementary Figure S2:** ACR20/50/70 responses (95% CI) to Week 52 by baseline MTX use in BE OPTIMAL, including reference (adalimumab) arm responses (NRI)
**Supplementary Figure S3:** PASI75/90/100 responses (95% CI) to Week 52 by baseline MTX use in BE OPTIMAL, including reference (adalimumab) arm responses (NRI)
**Supplementary Figure S4:** Additional composite efficacy outcomes (95% CI) to Week 52 by baseline MTX use in BE OPTIMAL, including reference (adalimumab) arm data (NRI)
**Supplementary Figure S5:** ACR 20/50/70 responses (with 95% CIs) to Week 52 by baseline MTX use (OC)
**Supplementary Figure S6:** PASI 75/90/100 responses (with 95% CIs) to Week 52 by baseline MTX use (OC)
**Supplementary Figure S7:** Additional composite efficacy outcomes (with 95% CIs) to Week 52 by baseline MTX use (OC)
**Supplementary Table S1.** Patient demographics and baseline characteristics for patients in the reference (adalimumab) arm of BE OPTIMAL
**Supplementary Table S2:** Additional efficacy endpoints (95% CI) at Week 52 by baseline MTX use (NRI, MI, WCI) for patients in the reference (adalimumab) arm of BE OPTIMAL
**Supplementary Table S3:** Safety outcomes to Week 52 for patients in the reference (adalimumab) arm of BE OPTIMAL
