# Correction to “Efficacy and Safety of Bimekizumab in Patients With Psoriatic Arthritis With or Without Methotrexate: 52‐Week Results From Two Phase 3 Studies”

**DOI:** 10.1002/acr2.70086

**Published:** 2025-07-09

McInnes, IB , Mease, PJ , Tanaka, Y et al. Efficacy and Safety of Bimekizumab in Patients With Psoriatic Arthritis With or Without Methotrexate: 52‐Week Results From Two Phase 3 Studies. ACROpen Rheumatol. 2024;6(11):720–731. doi: 10.1002/acr2.11727 PMC1155799039077886

In this article, the Disease Activity Index for Psoriatic Arthritis (DAPSA) disease states were calculated incorrectly across all treatment arms and timepoints, resulting in patients being wrongly allocated to a worse DAPSA disease state.

This error impacted three rows in Table 2. The corrected rows from Table 2 are provided below.

**Table:** Excerpt from Table 2:

	BE OPTIMAL (bDMARD‐naïve)				BE COMPLETE (TNFi‐IR)			
	Placebo (n = 281)		BKZ 160 mg Q4W (n = 431)		Placebo (n = 133)		BKZ 160 mg Q4W (n = 267)	
	+MTX, n = 163	−MTX, n = 118	+MTX, n = 252	−MTX, n = 179	+MTX, n = 51	−MTX, n = 82	+MTX, n = 119	−MTX, n = 148
DAPSA (WCI)								
LDA and REM,^a^ n (%)	113 (69.3)	72 (61.0)	170 (67.5)	109 (60.9)	25 (49.0)	41 (50.0)	69 (58.0)	88 (59.5)
REM, n (%)	42 (25.8)	31 (26.3)	91 (36.1)	67 (37.4)	12 (23.5)	13 (15.9)	42 (35.3)	44 (29.7)
95% CI	18.2 to 35.1	17.5 to 37.4	29.0 to 43.9	29.0 to 46.7	12.1 to 40.7	8.3 to 28.3	25.3 to 46.7	21.3 to 39.8

Randomized set. bDMARD, biologic disease‐modifying antirheumatic drug; BKZ, bimekizumab; CI, confidence interval; DAPSA, Disease Activity Index for Psoriatic Arthritis; LDA, low disease activity; +MTX, with methotrexate; −MTX, without methotrexate; Q4W, every four weeks; REM, remission; TNFi‐IR, prior inadequate response or intolerance to tumor necrosis factor inhibitors; WCI, worst‐category imputation.

^a^ Calculated by hand; 95% CIs are not available.

