# Supplementary material for: Efficacy and Safety of Bimekizumab in Patients With Psoriatic Arthritis With or Without Methotrexate: 52‐Week Results From Two Phase 3 Studies
Source: ACR Open Rheumatol. 2024 Jul 30;6(11):720–31. doi: 10.1002/acr2.11727 (PMC11557990; doi:10.1002/acr2.11727)
Supplement: Supplementary file 3 — Supplementary Figure S1: CONSORT diagrams for (A) BE OPTIMAL and (B) BE COMPLETE Supplementary Figure S2: ACR20/50/70 responses (95% CI) to Week 52 by baseline MTX use in BE OPTIMAL, including reference (adalimumab) arm responses (NRI) Supplementary Figure S3: PASI75/90/100 responses (95% CI) to Week 52 by baseline MTX use in BE OPTIMAL, including reference (adalimumab) arm responses (NRI) Supplementary Figure S4: Additional composite efficacy outcomes (95% CI) to Week 52 by baseline MTX use in BE OPTIMAL, including reference (adalimumab) arm data (NRI) Supplementary Figure S5: ACR 20/50/70 responses (with 95% CIs) to Week 52 by baseline MTX use (OC) Supplementary Figure S6: PASI 75/90/100 responses (with 95% CIs) to Week 52 by baseline MTX use (OC) Supplementary Figure S7: Additional composite efficacy outcomes (with 95% CIs) to Week 52 by baseline MTX use (OC) Supplementary Table S1. Patient demographics and baseline characteristics for patients in the reference (adalimumab) arm of BE OPTIMAL Supplementary Table S2: Additional efficacy endpoints (95% CI) at Week 52 by baseline MTX use (NRI, MI, WCI) for patients in the reference (adalimumab) arm of BE OPTIMAL Supplementary Table S3: Safety outcomes to Week 52 for patients in the reference (adalimumab) arm of BE OPTIMAL [file ACR2-6-720-s003.docx]

**Efficacy and safety of bimekizumab in patients with psoriatic arthritis with or without methotrexate: 52-week results from two phase 3 studies**

Iain B McInnes,^1^ Philip J Mease,^2^ Yoshiya Tanaka,^3^ Laure Gossec,^4,5^ M Elaine Husni,^6^ Lars Erik Kristensen,^7^ Richard B Warren,^8,9^ Barbara Ink,^10^ Rajan Bajracharya,^10^ Jason Coarse,^11^ Alice B Gottlieb^12^

*^1^College of Medical Veterinary and Life Sciences, University of Glasgow, Glasgow, UK; ^2^Swedish Medical Center/Providence St. Joseph Health and University of Washington, Seattle, WA, USA; ^3^The First Department of Internal Medicine, University of Occupational and Environmental Health, Japan, Kitakyushu, Fukuoka, Japan; ^4^Sorbonne Université, INSERM, Institut Pierre Louis d'Epidémiologie et de Santé Publique, Paris, France; ^5^AP-HP, Pitié‑Salpêtrière hospital, Rheumatology department, Paris, France; ^6^Department of Rheumatic and Immunologic Diseases, Cleveland Clinic, Cleveland, OH, USA; ^7^The Parker Institute, Copenhagen University Hospital, Bispebjerg and Frederiksberg, Denmark; ^8^Dermatology Centre, Northern Care Alliance NHS Foundation Trust, Manchester, UK; ^9^NIHR Manchester Biomedical Research Centre, Manchester University NHS Foundation Trust, Manchester Academic Health Science Centre, Manchester, UK; ^10^UCB Pharma, Slough, UK; ^11^UCB Pharma, Morrisville, NC, USA; ^12^Department of Dermatology, The Icahn School of Medicine at Mount Sinai, New York, NY, USA.*

**Correspondence to:** Iain B. McInnes, iain.mcinnes@glasgow.ac.uk

**Short title:** BE OPTIMAL & BE COMPLETE 1-Yr +/–MTX

SUPPLEMENTARY APPENDIX

Supplementary Figure S1. CONSORT diagrams for (A) BE OPTIMAL and (B) BE COMPLETE

1. BE OPTIMAL CONSORT diagram
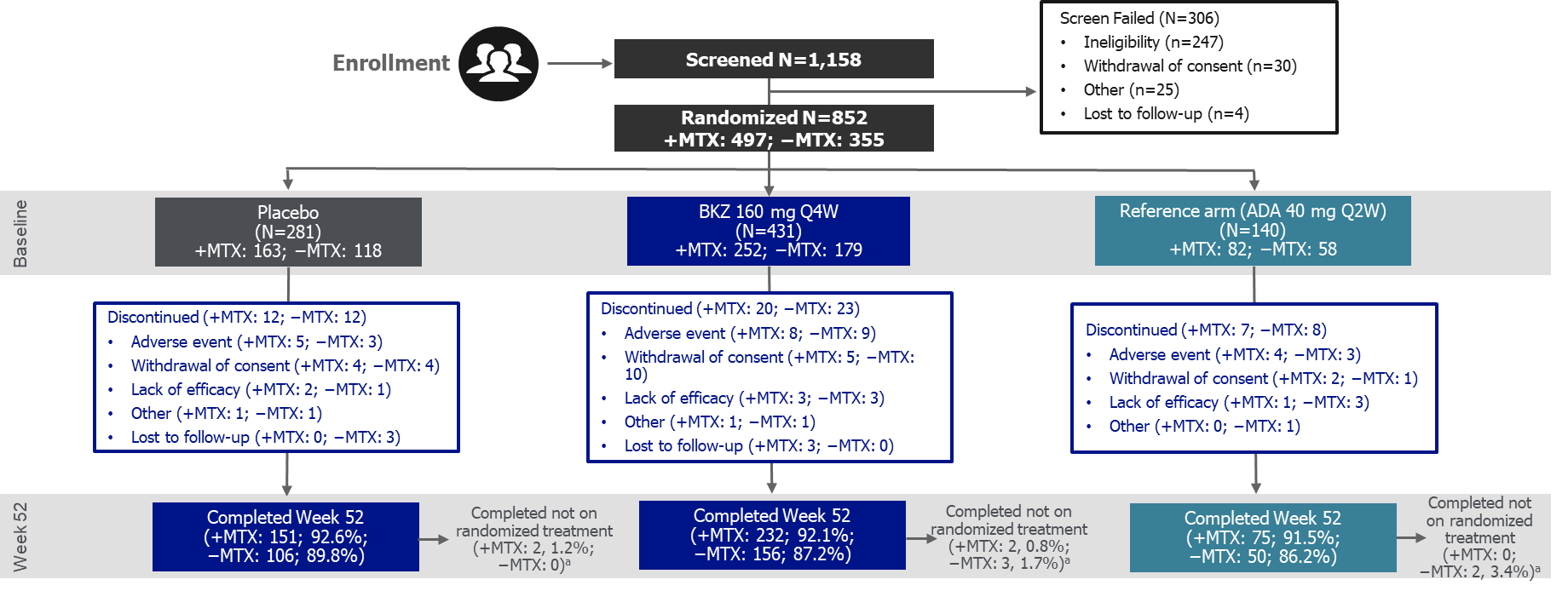

2. BE COMPLETE CONSORT diagram

**
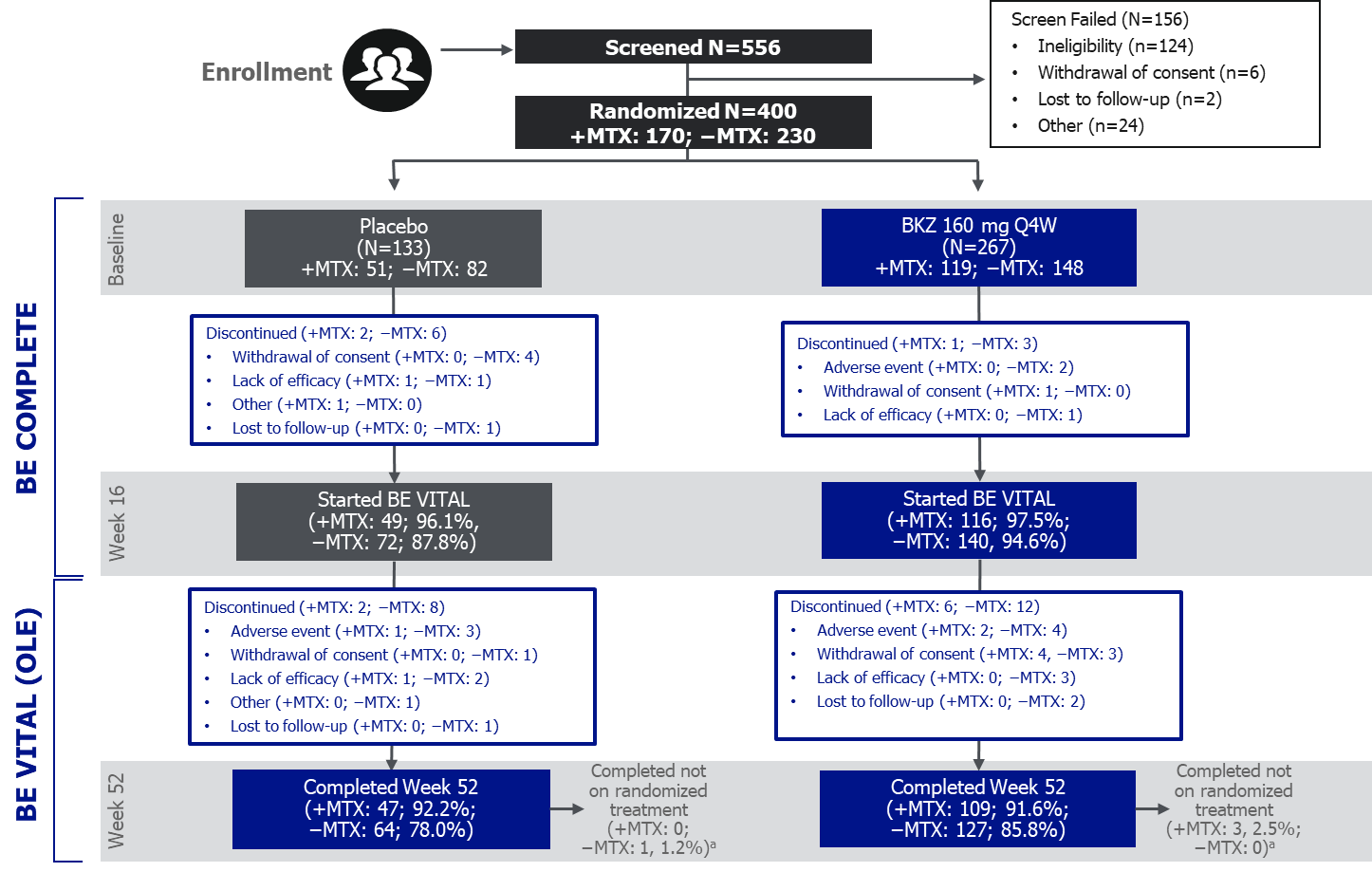
**

The percentage of patients completing or not completing treatment periods is calculated with the initial number of patients who were randomized to that group. In BE COMPLETE, 2 patients (1 +MTX, 1 –MTX) classified as ongoing as they did not have a visit for Week 52 but no formal discontinuation reason was reported. [a] Patients who withdrew from the study treatment or deviated from assigned randomized treatment but returned for all scheduled visits up to and including Week 52. ADA: adalimumab;
BKZ: bimekizumab; MTX: methotrexate; OLE: open label extension; Q2W: every two weeks; Q4W: every four weeks.

Supplementary Table S1. Patient demographics and baseline characteristics for patients in the reference (adalimumab) arm of BE OPTIMAL

|  | **BE OPTIMAL**  **(bDMARD‑naïve)** | |
| --- | --- | --- |
|  | **Reference arm**  **(ADA 40 mg Q2W)**  **n=140** | |
|  | **+MTX**  **n=82** | **−MTX**  **n=58** |
| Age, years, mean (SD) | 49.2 (11.7) | 48.8 (14.2) |
| Male, n (%) | 41 (50.0) | 30 (51.7) |
| BMI, kg/m^2^, mean (SD) | 28.4 (5.7) | 28.4 (6.2) |
| Time since first diagnosis of PsA, years, mean (SD) | 5.9 (6.2) | 6.5 (7.6)^a^ |
| Duration on MTX^b^, years, mean (SD) | 3.1 (5.1) | N/A |
| Weekly dose of MTX at baseline, mg, mean (SD) | 16.5 (5.1) | N/A |
| Tender joint count (of 68 joints), mean (SD) | 17.8 (13.1) | 17.2 (13.1) |
| Swollen joint count (of 66 joints), mean (SD) | 9.8 (7.4) | 9.4 (6.7) |
| hs-CRP, mg/L, mean (SD) | 6.9 (10.8) | 6.2 (10.3) |
| Psoriasis with ≥3% BSA, n (%) | 37 (45.1) | 31 (53.4) |
| PASI score,^c^ mean (SD) | 9.6 (8.1) | 7.3 (6.8) |
| Presence of enthesitis (LEI >0), n (%)  Score,^d^ mean (SD) | 18 (22.0)^a^ 2.2 (1.6)^a^ | 18 (31.0) 2.3 (1.6) |
| Presence of dactylitis (LDI >0), n (%)  Score,^e^ mean (SD) | 5 (6.1)^a^ 54.1 (37.3)^a^ | 6 (10.3) 46.0 (29.8) |
| Presence of nail psoriasis (mNAPSI >0), n (%)  Score,^f^ mean (SD) | 42 (51.2) 3.7 (2.2) | 33 (56.9) 3.8 (2.4) |
| HAQ-DI score, mean (SD) | 0.91 (0.55) | 0.79 (0.53) |
| Patient’s assessment of pain VAS score, mean (SD) | 56.5 (23.9) | 57.0 (24.2) |
| Physician’s global assessment score, mean (SD) | 58.0 (17.2) | 56.2 (17.9)^a^ |
| Patient’s global assessment score, mean (SD) | 57.3 (21.8) | 56.7 (22.0) |

Randomized set. [a] Data missing for 1 patient; [b] Prior to first administration of study drug; [c] In patients with ≥3% BSA affected by psoriasis at baseline; [d] In patients with an LEI score >0 at baseline; [e] In patients with an LDI score >0 at baseline; [f] In patients with a mNAPSI score >0 at baseline. ADA: adalimumab; bDMARD: biologic disease-modifying antirheumatic drug; BMI: body mass index; BSA: body surface area; HAQ-DI: Heath Assessment Questionnaire – Disability Index; hs‍-‍CRP:‍ high sensitivity C-reactive protein; LDI: Leeds Dactylitis Index; LEI: Leeds Enthesitis Index; mNAPSI: modified Nail Psoriasis Severity Index; MTX:‍ methotrexate; N/A: not applicable; PASI:‍ Psoriasis Area and Severity Index; PsA:‍ psoriatic arthritis; Q2W: every 2 weeks; SD: standard deviation; VAS:‍ visual analog scale.

Supplementary Table S2. Additional efficacy endpoints (95% CI) at Week 52 by baseline MTX use (NRI, MI, WCI) for patients in the reference (adalimumab) arm of BE OPTIMAL

|  | **BE OPTIMAL**  **(bDMARD‑naïve)** | |
| --- | --- | --- |
|  | **Reference arm**  **(ADA 40 mg Q2W)**  **n=140** | |
|  | **+MTX**  **n=82** | **−MTX**  **n=58** |
| Enthesitis resolution,^a^ n/N (%) | 11/18 (61.1) [38.6, 83.6] | 10/18 (55.6) [32.6, 78.5] |
| Dactylitis resolution,^b^ n/N (%) | 4/5 (80.0) [44.9, 100] | 4/6 (66.7) [29.0, 100] |
| Nail psoriasis resolution,^c^ n/N (%) | 24/42 (57.1) [42.2, 72.1] | 21/33 (63.6) [47.2, 80.1] |
| HAQ-DI CfB [MI], mean (SE) | –0.49 (0.06) [–0.61, –0.37] | –0.30 (0.08) [–0.45, –0.14] |
| DAPSA [WCI] |  |  |
| LDA+REM^d^  REM | 50 (61.0)  27 (32.9) [21.6, 46.7] | 24 (41.4)  14 (24.1) [13.1, 40.2] |

Randomized set. NRI unless otherwise stated. [a] In patients with an LEI score >0 at baseline;
[b] In patients with an LDI score >0 at baseline; [c] In patients with an mNAPSI score >0 at baseline;
[d] Calculated by hand, 95% CIs not available. ADA:‍ adalimumab; bDMARD: biologic disease‑modifying antirheumatic drug; CfB:‍ change from baseline; DAPSA: Disease Activity Index for Psoriatic Arthritis; HAQ-DI: Heath Assessment Questionnaire – Disability Index; LDA: low disease activity; LDI: Leeds Dactylitis Index; LEI: Leeds Enthesitis Index; MI:‍ multiple imputation; mNAPSI: modified Nail Psoriasis Severity Index; MTX:‍ methotrexate; NRI:‍ non‑responder imputation; Q2W: every 2 weeks; REM:‍ remission; SE:‍ standard error; WCI: worst-category imputation.

Supplementary Figure S2. ACR20/50/70 responses (95% CI) to Week 52 by baseline MTX use in BE OPTIMAL, including reference (adalimumab) arm responses (NRI)
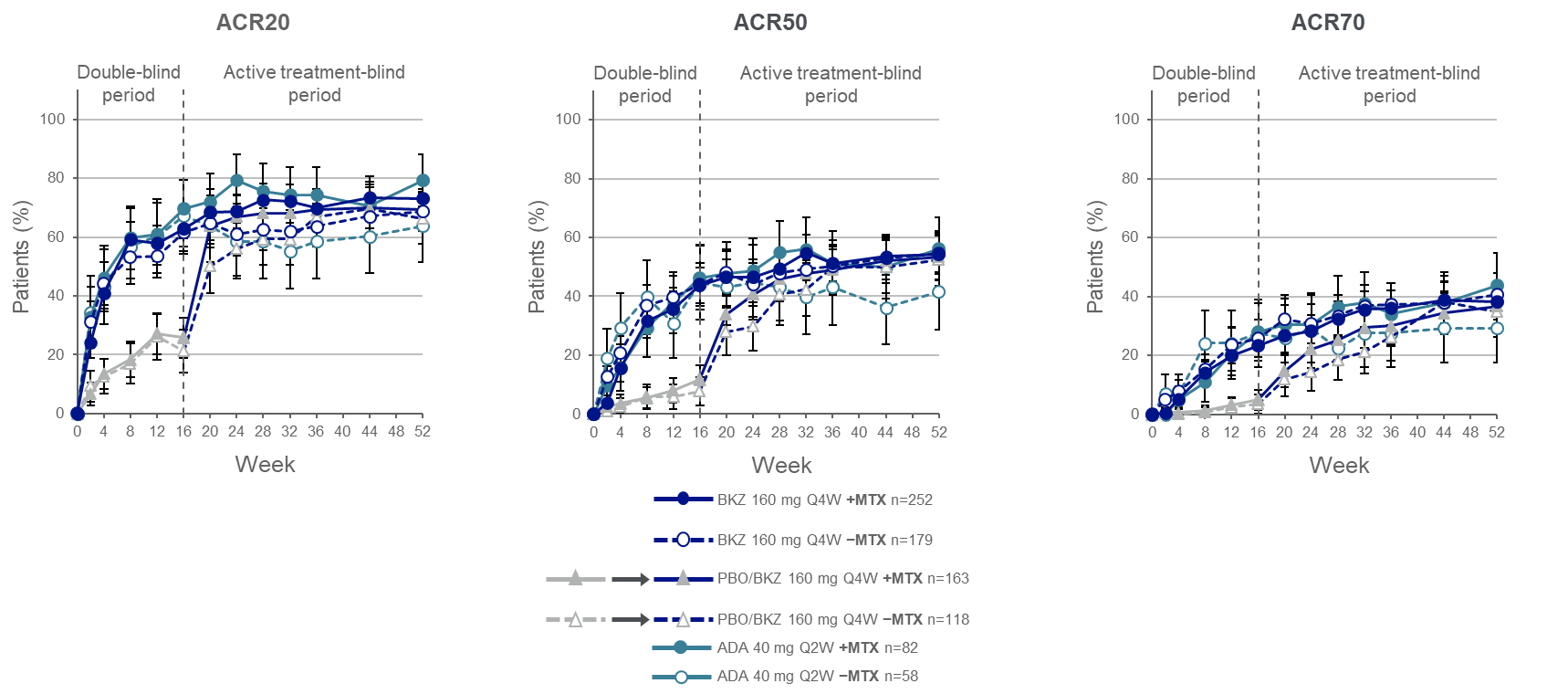


Randomized set. Only data from BE OPTIMAL (bDMARD-naïve) shown. Error bars show 95% CIs. A 95% CI was not able to be evaluated for patients randomized to PBO at Week 4 in ACR70. ACR20/50/70: ≥20%/50%/70% improvement in American College of Rheumatology response criteria; ADA: adalimumab; bDMARD: biologic disease‑modifying antirheumatic drug; BKZ: bimekizumab; CI: confidence interval; MTX: methotrexate; PBO: placebo; Q2W:‍ every 2 weeks; Q4W: every 4 weeks.

Supplementary Figure S3. PASI75/90/100 responses (95% CI) to Week 52 by baseline MTX use in BE OPTIMAL, including reference (adalimumab) arm responses (NRI)
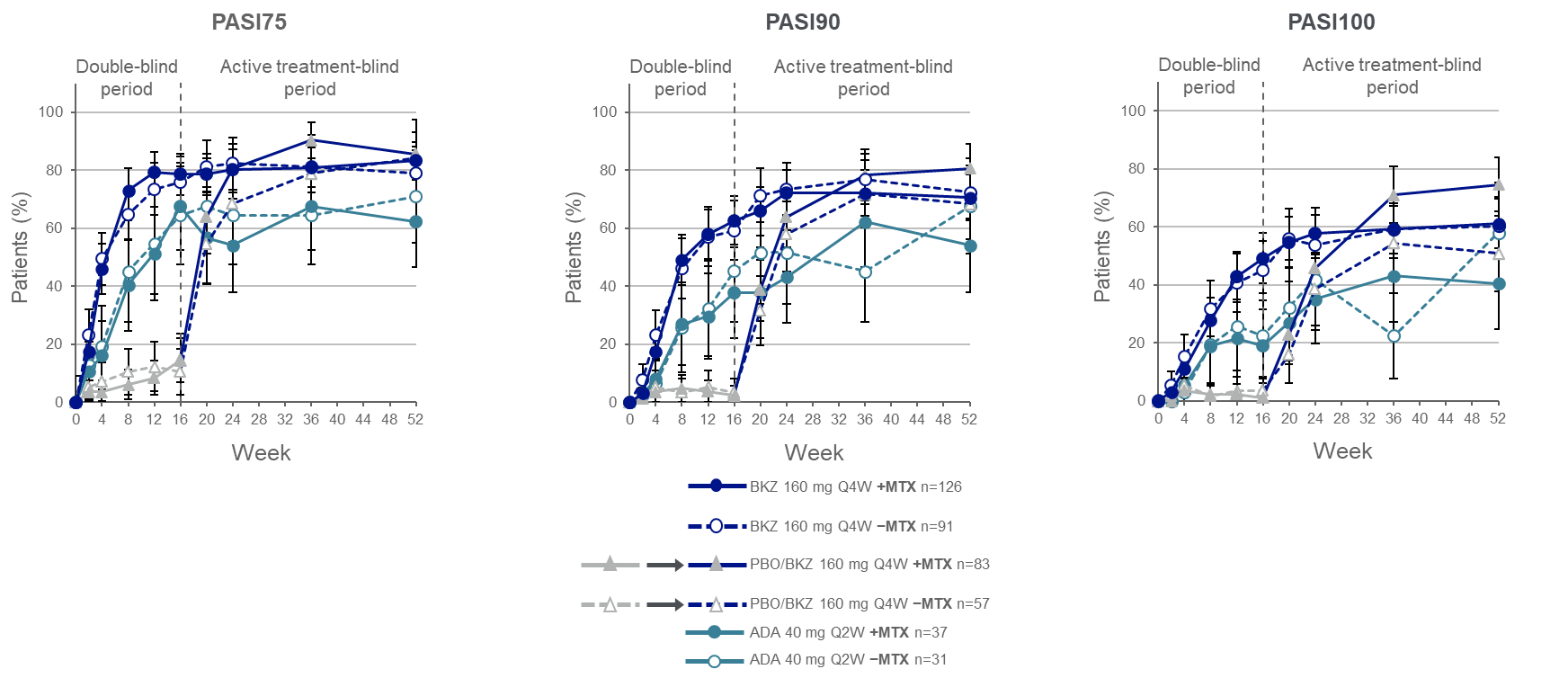


Randomized set. Only data from BE OPTIMAL (bDMARD-naïve) shown. Error bars show 95% CIs. A 95% CI was not able to be evaluated for patients randomized to PBO at Week 2 in PASI100. ADA: adalimumab; bDMARD: biologic disease-modifying antirheumatic drug; BKZ: bimekizumab; CI: confidence interval; MTX: methotrexate; NRI:‍ non-‍responder imputation; PASI75/90/100: ≥75%/90%/100% improvements in Psoriasis Area and Severity Index; PBO: placebo; Q2W: every 2 weeks; Q4W: every 4 weeks.

Supplementary Figure S4. Additional composite efficacy outcomes (95% CI) to Week 52 by baseline MTX use in BE OPTIMAL, including reference (adalimumab) arm data (NRI)
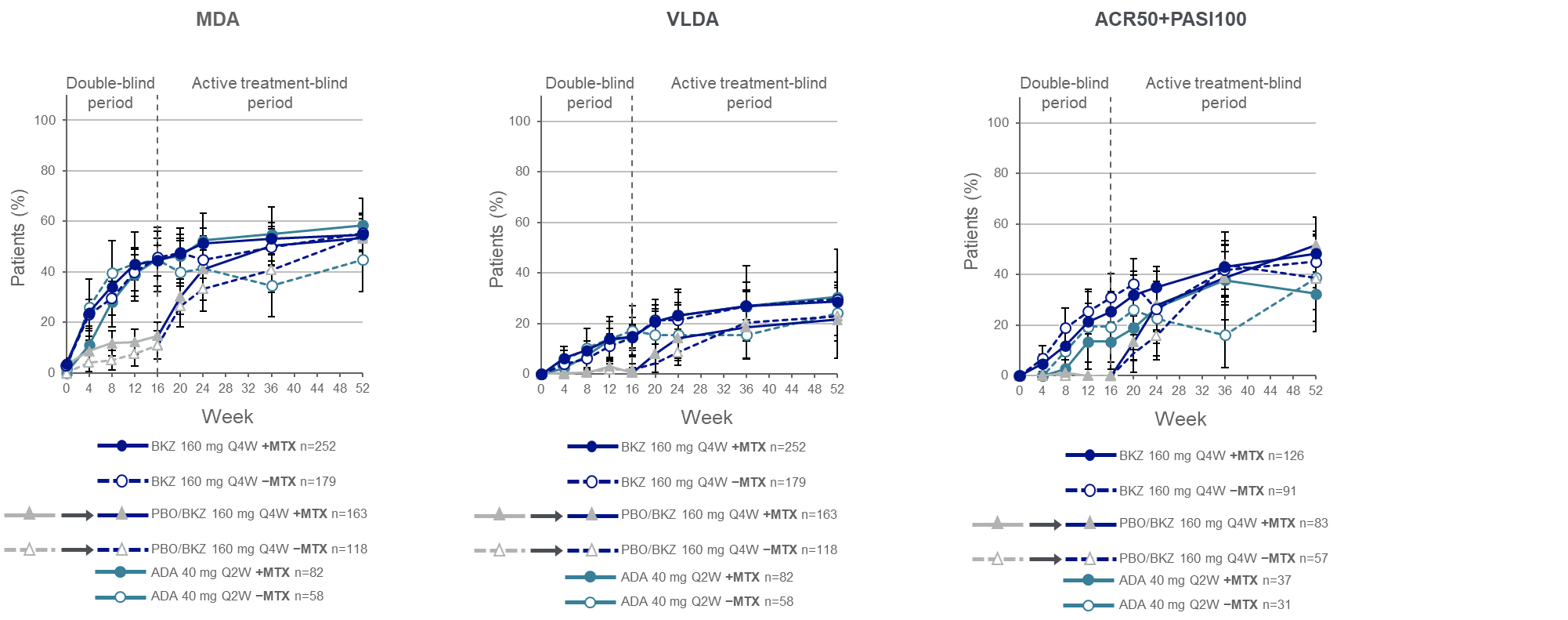


Randomized set. Only data from BE OPTIMAL (bDMARD-naïve) shown. Error bars show 95% CIs. ACR50+PASI100 data in patients with psoriasis BSA ≥3% at baseline.
ACR50+PASI100: Achievement of both ≥50% improvement in American College of Rheumatology response criteria and 100% improvement in Psoriasis Area and Severity Index; ADA: adalimumab; bDMARD: biologic disease-modifying antirheumatic drug; BKZ: bimekizumab; CI:‍ confidence interval; MDA: minimal disease activity;
MTX: methotrexate; NRI: non-responder imputation; PBO: placebo; Q2W: every 2 weeks; Q4W: every 4 weeks; VLDA: very low disease activity.

Supplementary Figure S5. ACR 20/50/70 responses (with 95% CIs) to Week 52 by baseline MTX use (OC)
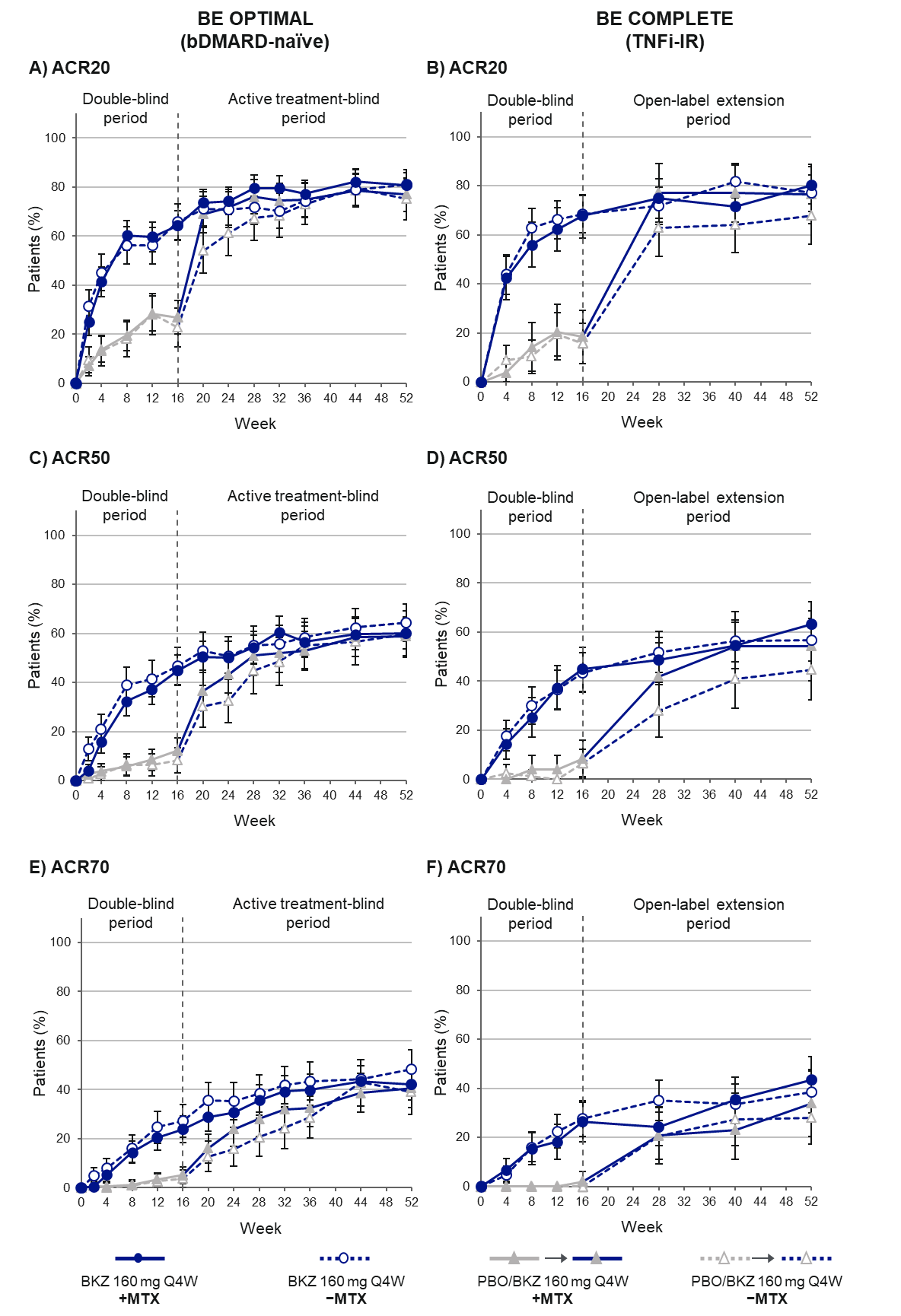


Randomized set. Error bars show 95% CIs. A 95% CI was not able to be evaluated for patients randomized to PBO at Week 4 in ACR70. BE ACR20/50/70: ≥20%/50%/70% improvement in American College of Rheumatology response criteria; bDMARD: biologic disease-modifying antirheumatic drug; BKZ: bimekizumab; CI: confidence interval; MTX: methotrexate; PBO: placebo; Q4W: every 4 weeks; TNFi-IR: prior inadequate response or intolerance to tumor necrosis factor inhibitors.

Supplementary Figure S6. PASI 75/90/100 responses (with 95% CIs) to Week 52 by baseline MTX use (OC)
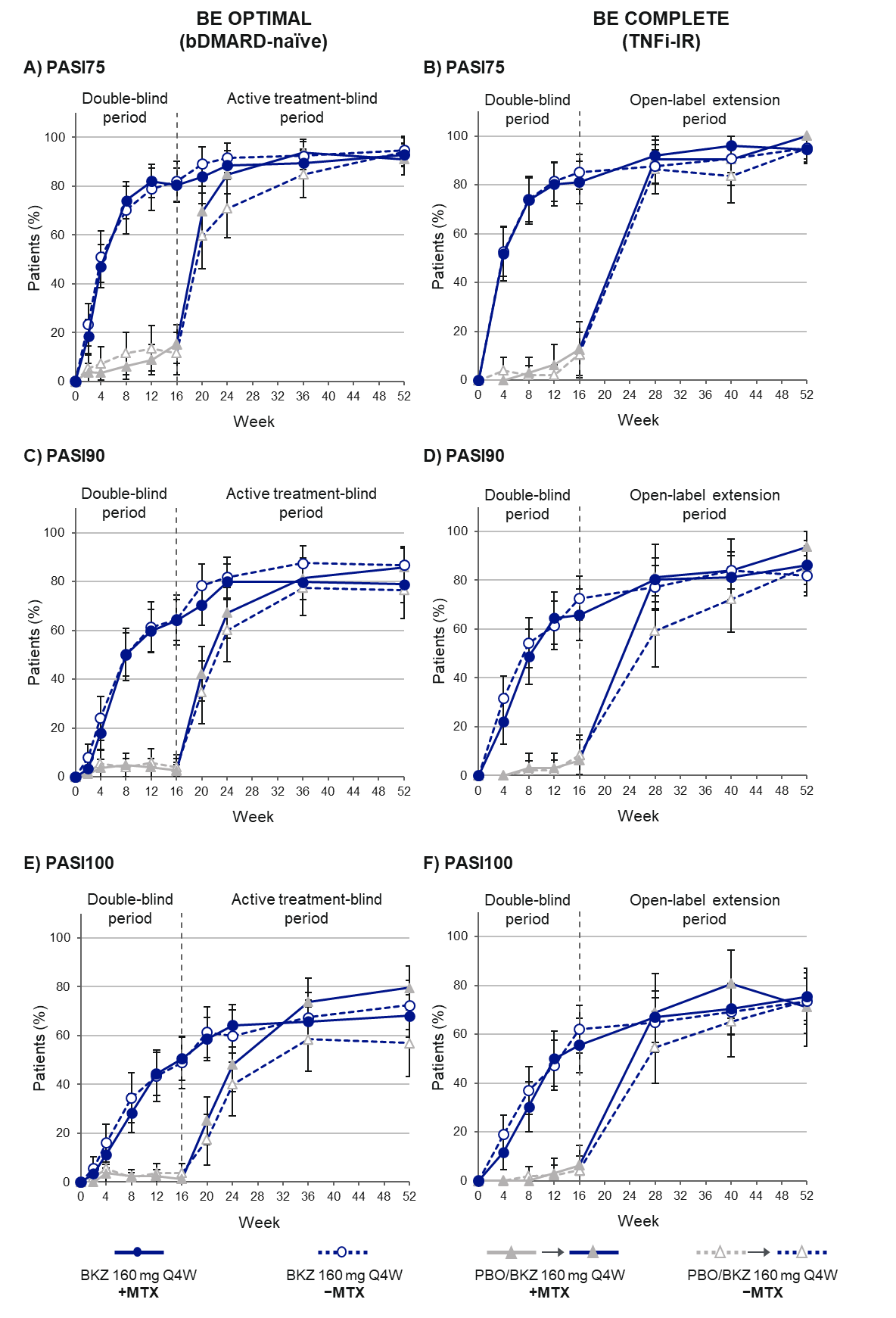


Randomized set, in patients with ≥3% body surface area affected by psoriasis at baseline. Error bars show 95% CIs. A 95% CI was not able to be evaluated for patients randomized to PBO at Week 2 in PASI100. bDMARD: biologic disease-modifying antirheumatic drug; BKZ: bimekizumab; CI:‍ confidence interval; MTX: methotrexate; PASI75/90/100: ≥75%/90%/100% improvements in Psoriasis Area and Severity Index; PBO: placebo; Q4W: every 4 weeks; TNFi-IR: prior inadequate response or intolerance to tumor necrosis factor inhibitors.

Supplementary Figure S7. Additional composite efficacy outcomes (with 95% CIs) to Week 52 by baseline MTX use (OC)
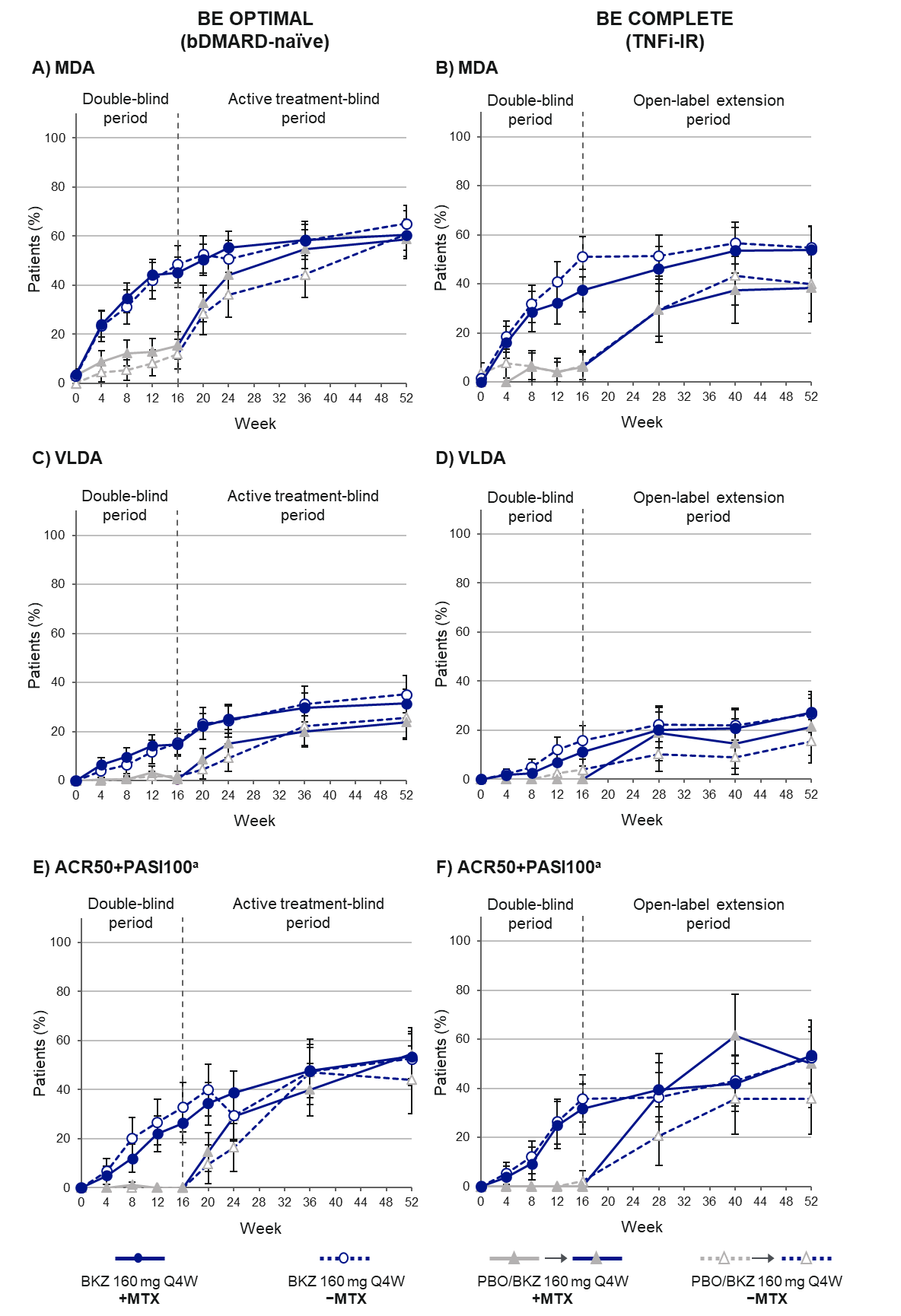


Randomized set. Error bars show 95% CIs. [a] ACR50+PASI100 in patients with psoriasis BSA ≥3% at baseline. ACR50+PASI100: Achievement of both ≥50% improvement in American College of Rheumatology response criteria and 100% improvement in Psoriasis Area and Severity Index; bDMARD:‍ biologic disease-modifying antirheumatic drug; BKZ: bimekizumab; CI: confidence interval; MDA: minimal disease activity; MTX: methotrexate; PBO: placebo; Q4W: every 4 weeks; TNFi-IR: prior inadequate response or intolerance to tumor necrosis factor inhibitors; VLDA: very low disease activity.

Supplementary Table S3. Safety outcomes to Week 52 for patients in the reference (adalimumab) arm of BE OPTIMAL

|  | **BE OPTIMAL**  **(bDMARD‑naïve)** | | |
| --- | --- | --- | --- |
|  | **Reference arm**  **(ADA 40 mg Q2W)**  **n=140** | | |
| **n (%) [EAIR/100 PY]^a^** | **+MTX**  **n=82**  **PYs: 80.7** | **−MTX**  **n=58**  **PYs: 56.1** | |
| Any TEAE | 63 (76.8) [169.2] | 50 (86.2) [298.9] | |
| Serious TEAEs | 7 (8.5) [9.0] | 3 (5.2) [5.4] | |
| Study discontinuation due to TEAE | 4 (4.9) [5.1] | 3 (5.2) [5.5] | |
| Drug-related TEAEs^b^ | 30 (36.6) | 24 (41.4) | |
| Severe TEAEs | 7 (8.5) | 2 (3.4) | |
| Deaths | 0 | 0 | |
| Most frequent TEAEs^c^ |  |  | |
| Nasopharyngitis | 3 (3.7) [3.8] | 9 (15.5) [18.1] | |
| Upper respiratory tract infection | 4 (4.9) [5.1] | 4 (6.9) [7.5] | |
| Urinary tract infection | 2 (2.4) [2.5] | 3 (5.2) [5.5] | |
| Headache | 4 (4.9) [5.1] | 2 (3.4) [3.6] | |
| Oral candidiasis | 1 (1.2) [1.3]^d^ | 0 | |
| Diarrhea | 2 (2.4) [2.5] | 5 (8.6) [9.5] | |
| Pharyngitis | 3 (3.7) [3.8] | 0 | |
| SARS-CoV-2 (COVID-19) | 1 (1.2) [1.2] | 3 (5.2) [5.4] | |
| Uveitis | 0 | 0 | |
| Adjudicated MACE | 0 | 0 | |
| Neutropenia^e^ | 2 (2.4) [2.5] | 0 | |
| Infections |  | |  |
| Serious | 2 (2.4) [2.5] | 0 | |
| Opportunistic | 1 (1.2) [1.3] | 0 | |
| Hypersensitivity^f^ | 4 (4.9) [5.1] | 3 (5.2) [5.6] | |
| Dermatitis and eczema | 2 (2.4) [2.5] | 0 | |
| Injection site reactions | 5 (6.1) [6.5] | 8 (13.8) [15.9] | |
| Adjudicated suicidal ideation and behavior | 0 | 0 | |
| Liver function test changes/enzyme elevations |  | |  |
| ALT >3 × ULN | 4/82 (4.9) | 3/57 (5.3) | |
| AST or ALT >3 × ULN | 5/82 (6.1) | 4/57 (7.0) | |
| Adjudicated IBD^g^ | 0 | 0 | |
| Malignancies excluding nonmelanoma skin cancer | 0 | 0 | |
| Nonmelanoma skin cancer |  |  | |
| Skin neoplasm | 0 | 1 (1.7) [1.8] | |

Safety set. [a] EAIRs are reported where available; [b] As assessed by the reporter; [c] Most frequent adverse events are those occurring in ≥5% of BKZ-treated patients in BE OPTIMAL or BE COMPLETE, reported for the reference arm in BE OPTIMAL; [d] The infection was mild; [e] Both neutropenia cases; [f] No cases were serious; [g] Including define or probable TEAEs. ALT: alanine aminotransferase; AST: ‍aspartate aminotransferase; bDMARD:‍ biologic disease‑modifying antirheumatic drug; SARS‑CoV‑2/COVID‑19:‍ coronavirus 2019; EAIR:‍ exposure‑adjusted incidence rate; IBD: inflammatory bowel disease; MACE: major adverse cardiovascular event; MTX:‍ methotrexate; PY: patient year; Q2W:‍ every 2 weeks; TEAE:‍ treatment‑emergent adverse event; ULN: upper limit of normal.
